# Transcranial magnetic stimulation effects on cognitive enhancement in mild cognitive impairment and Alzheimer's disease: a systematic review and meta-analysis

**DOI:** 10.3389/fneur.2023.1209205

**Published:** 2023-07-17

**Authors:** Yixin Yan, Minjie Tian, Tong Wang, Xixi Wang, Yingying Wang, Jingping Shi

**Affiliations:** Department of Neurology, Affiliated Nanjing Brain Hospital, Nanjing Medical University, Nanjing, China

**Keywords:** transcranial magnetic stimulation, Alzheimer's disease, mild cognitive impairment, cognition, meta-analysis, cerebellum

## Abstract

**Introduction:**

Transcranial magnetic stimulation (TMS) is a non-invasive intervention that holds promise for improving cognitive function in individuals with Alzheimer's disease (AD). However, the effectiveness of this therapy and the optimal TMS parameters has not reached a consensus. The purpose of the meta-analysis was to systematically discern the effectiveness of different components of TMS protocols on cognitive improvement in patients with mild cognitive impairment (MCI) and AD.

**Methods:**

The meta-analysis was preregistered on Prospero (registration number: CRD42022345482). PubMed, Web of Science, Science Direct, and Cochrane Library databases were used to search, screen and identify eligible studies with the following keywords: Transcranial Magnetic Stimulation OR TMS OR theta burst stimulation AND Alzheimer OR Alzheimers OR Alzheimer's OR mild cognitive impairment OR MCI. Randomized controlled trials (RCTs) of participants with accepted standardized diagnostic criteria were searched by two authors independently. The risk of bias was assessed using an adapted Cochrane Risk of Bias tool. Standardized mean difference (SMD) and 95% confidence interval (CI) were calculated using the random-effects models. Subgroup analyses were performed to investigate the influential factors.

**Results:**

A total of 21 studies and 25 trials were included in this meta-analysis. The findings revealed a significant overall cognition improvement of real stimulation compared with sham stimulation (short-term effects: SMD, 0.91; 95% CI 0.44–1.38; *P* < 0.01; long-lasting effects: SMD, 0.91; 95% CI 0.27–1.55; *P* < 0.01). Subgroup analysis demonstrated that stimulation of the left dorsolateral prefrontal cortex and bilateral cerebellums, as well as moderate frequency stimulation (5 Hz and 10 Hz) on mild and moderate cognitive impairment patients, were more effective than other TMS protocols. However, the additional application of cognitive training showed no significant improvement.

**Conclusion:**

Cognitive improvement effect of TMS was demonstrated in MCI and AD patients in both short-term assessment and long-lasting outcomes, and the efficiency of TMS is affected by the stimulation frequency, stimulation site, and participant characteristics. Further RCTs are needed to validate the findings of our subgroup analysis.

**Systematic review registration:**

https://www.crd.york.ac.uk/prospero/display_record.php?ID=CRD42022345482, identifier: CRD42022345482.

## Introduction

Alzheimer's disease is the most prevalent neurodegenerative disease, characterized by progressive deterioration of memory and other cognitive function, accompanied by abnormal neuropsychiatric behavior (1). AD pose a substantial healthcare challenge globally, affecting ~4% of the elderly population worldwide up to 2025 and developing in an estimated 6, 7 million people annually (2). However, currently approved clinical treatments for AD have limited efficacy (3) and development of pharmacological interventions has faced significant challenges over the past two decades (4). Consequently, novel therapeutic approaches have gained increasing attention. and non-invasive electrical brain stimulation (NIBS) has emerged as a potential alternative (5).

Transcranial magnetic stimulation (TMS), the most common form of NIBS, modulates cortical excitability and neuroplasticity by inducing electromagnetic pulses to the brain (6, 7). TMS can be classified into various forms according to the frequency and interval of stimulation. Among these, conventional low and high frequency repetitive transcranial magnetic stimulation (rTMS) and patterned rTMS, such as theta-burst stimulation (TBS) (8), are the most commonly used for therapeutic purposes and serve as the primary focus of our research. Previous studies have demonstrated that high-frequency (HF) rTMS (>1 Hz) or intermittent theta-burst stimulation induce an enhanced effect, whereas low-frequency (LF) rTMS ( ≤ 1 Hz) or continuous theta-burst stimulation suppress neural activity (9, 10). TMS has proven its safety and barely has contraindications (11), allowing for its widespread clinical application in the treatment of patients with mild cognitive impairment (MCI) and AD. However, the precise therapeutic effects and optimal TMS parameters remain debatable and thus necessitate further research. Therefore, this meta-analysis aimed to systematically analyze the effectiveness of different components of TMS protocols in enhancing cognitive function in patients with MCI and AD.

To date, 15 meta-analyses have summarized the effects of rTMS on patients with AD or MCI. Hovever, none of these meta-analyses included TBS as the treatment modality. Among the existing meta-analyses, five primarily focused on comparing the therapeutic effects of rTMS with other non-invasive interventions, such as transcranial direct current stimulation (tDCS) and cognitive training (CT) (12–16). These studies reported effective results for rTMS and controversial effects for DCS and CT in AD patients. Five studies evaluated the effects of different stimulation sites on the efficacy of rTMS. Most of these studies compared the effects of left and right dorsolateral prefrontal cortex (DLPFC) stimulation or DLPFC stimulation with other brain regions (17–20). Only one study explored the effects of specific brain regions, comparing memory and general cognition improvement in the DLPFC and temporo-parietal regions (21), concluded significant memory improvement only in the DLPF. However, these studies have yielded inconsistent conclusions and lack a comprehensive comparison of stimulation sites.

The present meta-analysis was strictly based on randomized controlled trials (RCTs) and included 21 studies (25 trials), surpassing the number of previous studies (The minimum number of included TMS studies was five and the maximum was 13). Notably, we incorporated one study that investigated a novel stimulation site not previously explored in AD patients (22). In addition, we performed comprehensive subgroup analyses considering stimulation parameters, trial designs and beneficiary groups to elucidate the appropriate TMS protocols and provide guidance for the clinical application of TMS in AD treatment.

## Materials and methods

### Search strategy

The meta-analysis was preregistered on Prospero (registration number: CRD42022345482, Available from: https://www.crd.york.ac.uk/prospero/display_record.php?ID=CRD42022345482) and conducted on October 1, 2022, using the PubMed, Web of Science, Science Direct, and Cochrane Library databases with the following keywords: Transcranial Magnetic Stimulation OR TMS OR theta burst stimulation AND Alzheimer OR Alzheimers OR Alzheimer's OR mild cognitive impairment OR MCI.

### Inclusion and exclusion criteria

Two investigators (YY and TW) independently searched for RCTs that compared active TMS treatment with sham treatment in patients diagnosed with AD or MCI, based on accepted standardized diagnostic criteria. The inclusion criteria were as follows: (1) participants diagnosed with AD or MCI according to accepted standardized criteria (e.g.,—DSM, NIAAA, NINCDS-ADRDA, or Petersen's criteria for MCI); (2) presence of a sham-controlled condition with either parallel or cross-over design; (3) outcome measures based on cognitive function assessments; (4) studies published in English; and (5) studies limited to human subjects. The exclusion criteria were as follows: (1) non-primary studies, such as reviews, meta-analyses, editorials, conference abstracts, case studies, and protocols; (2) absence of TMS-sham-controlled groups; (3) TMS not intended as a treatment; (4) cognitive impairment due to non-AD conditions (e.g., Parkinson's disease, stroke); and (5) unavailability of the necessary data.

### Data extraction

Two authors (YY and YW) independently extracted the data using a predesigned data extraction form, and any discrepancies were resolved through consensus. The extracted data included sample size, age, sample characteristics, TMS protocol, cognitive performance outcomes, and timing of the outcome assessments. In cases where the mean and standard deviation (SD) of cognitive outcomes were not provided directly, the corresponding authors were contacted or the values were calculated using formulas from the Cochrane Handbook 5.1.0, Chapter 16.1.3.2 (23). The Coefficient refers to the correlation coefficients, and the value Coefficient was imputed from another study (22) with complete data included in this meta-analysis (23), where the coefficient value was determined to be 0.8.


Mean change = mean final−mean baseline 

$$\begin{array}{c} SD\;change\;=\;\\ \sqrt{SD\;baseline^{2}\;+\;SD\;final^{2}-(2\times Coefficent\times SD\;baseline\;\times \;SD\;final}) \end{array}$$

### Evaluation of risk of bias

The risk of bias was assessed using items adapted from the Cochrane Risk of Bias tool (24). The assessment criteria included (1) use of accepted standardized criteria for AD/MCI diagnosis; (2) specific methods for random sequence generation; (3) blinding of personnel and participants; 4) blinding of outcome assessment; (5) similarity of characteristics between the active and sham groups; and (6) reporting of participant dropout numbers. Each study was assigned a quality score, with a score of 1 indicating compliance with the assessment, 0 indicating non-compliance, and “un” indicating that the information was not reported. A higher score indicates better quality. The methodological quality of each included study was independently assessed by two authors (YY and MT) and any disagreements were resolved through discussion.

### Statistical analysis

Statistical analysis was performed using the Stata 16.0 statistical software (Stata Corp., College Station, TX, USA). The standardized mean difference (SMD) and 95% confidence interval (CI) were calculated to summarize the effect size of the clinical scores pre- and post-treatment in the experimental and control groups. We preferred using Hedges's g (25) for SMD estimation because of its reduced bias in small sample sizes. The DerSimonian-Laird method was used to synthesize SMD estimates (26). The heterogeneity of the included studies was assessed using Cochran's Q statistic and the I^2^ test. The random-effects model was applied to obtain a more conservative result.

### Subgroup analyses

Additional subgroup comparisons were conducted to determine appropriate TMS protocols. we compared both short term and long-lasting efficacy of TMS in the following categories: (1) stimulation sites including the left and bilateral DLPFC, parietotemporal area, bilateral cerebellum and precuneus; (2) TMS frequency including 1 Hz, 5 Hz, 10 Hz, 20 Hz, and 50 Hz iTBS; (3) TMS with or without cognitive training; and 4) patients with mild, mederate or severe cognitive impairment.

## Results

### Information on the included studies

**Figure 1:**
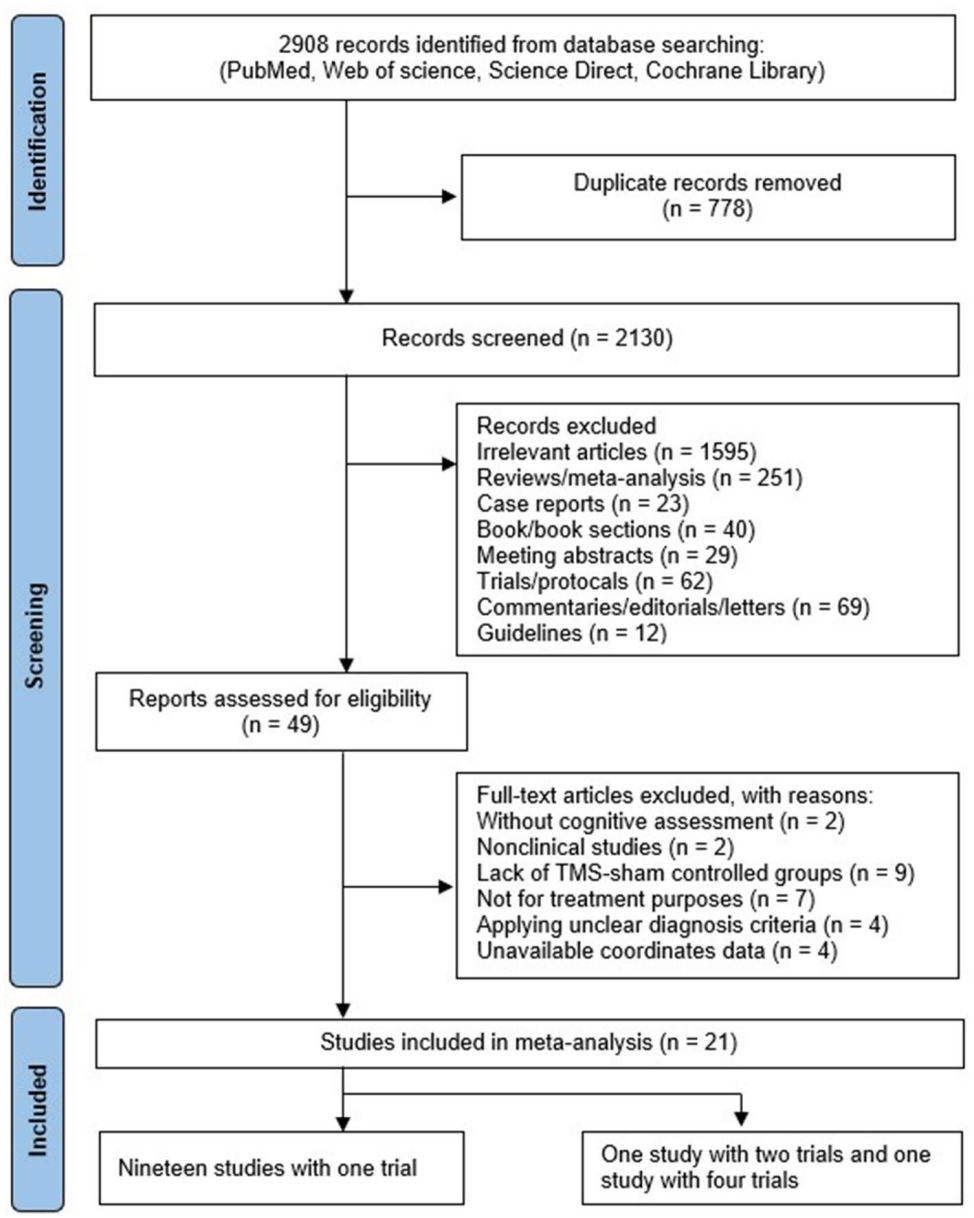
Flow diagram summarizing the selection process.

A total of 2,908 studies were initially identified through the primary search. After removing duplicates (*n* = 778), irrelevant studies (*n* = 1,595), and non-RCT studies (*n* = 486), 49 studies remained for the full-text screening. Following the application of exclusion criteria, including studies without cognitive assessment (*n* = 2), non-clinical studies (*n* = 2), lack of TMS-sham-controlled groups (*n* = 9), studies not intended for treatment (*n* = 7), unclear diagnostic criteria (*n* = 4), and unavailable data (*n* = 4),a total of 21 studies (25 trials) involving 806 MCI and AD patients were included in this analysis. A flow diagram illustrating the study selection process was conducted according the PRISMA statement (Figure 1).

Among the included studies, Most studies reported only one trial, with one reported two trials (27) and one reported four trials (28). Eighteen applied a parallel design (22, 27–41), whereas three studies utilized a crossover patterns (42–44). The majority of studies employed moderate-to high-frequency rTMS, with one study using low-frequency rTMS (LFrTMS) (28) and one using intermittent theta-burst stimulation (iTBS) (35). Single site TMS was applied in 11 studies, including left dorsolateral prefrontal cortex (L-DLPFC) (30–33, 35, 38, 40, 42, 43) and the left inferior parietal lobule (37, 45), Four studies used bilateral stimulation (22, 28, 44, 46) and six studies employed multiple stimulation sites: Broca and Wernicke area (29, 36, 39, 41), bilateral dorsolateral prefrontal cortex (DLPFC) (29, 36, 39, 41), bilateral parietal somatosensory association cortex (pSAC) (36, 41), bilateral inferior parietal lobule (IPL) (29, 39), parietal and posterior temporal area (27), and L-DLPFC and left lateral temporal lobe (LTL) (34). Cognitive outcomes assessed in these studies studies included Mini Mental Status Examination (MMSE) (22, 28, 30–32, 35, 37, 40, 43, 44), Alzheimer's disease Assessment Scale-Cognitive (ADAS-cog) (29, 34, 36, 39, 41, 47), Montreal Cognitive Assessment (MOCA) (38, 42), and Rivermead Behavioral Memory Test (RBMT) (33). The basic characteristics of these articles are summarized in Table 1.

**Table 1:** The basic characteristics of the included studies.

**Study name**	**Sample size**	**Mean age**	**Female**	**Baseline MMSE^6^**	**Cognitive impairment level**	**TMS Target**	**TMS protocal**	**Intensity**	**Pluses**	**Dur-ation**	**Sham condition**
	**Real/sham**	**Real/sham**	**Real/sham**	**Real/sham**							
Wei et al. (45)	29/27	70.0/71.7	20/20	14.5/13.7	Moderate	Left lateral parietal	10 hz rTMS	100–110% RMT	800 x 10	2 w	Rotated 45°
Koch et al. (46)	25/25	75.0/72.3	14/12	21.2/21.5	Mild	Bilateral precuneus	20 hz rTMS	100%RMT	1,600 x 32	2 w+22 w	Sham coil
Wu et al. (35)	24/23	66.5/66.4	14/12	20.5/21.7	Moderate	Left DLPFC^1^	50hz iTBS	70%RMT	1,800 x 14	2 w	A placebo coil
Vecchio et al. (36)	30/17	71.1/72.2	16/7	22.9/20.6	Mild	Broca's area, R-DLPFC^1^ and L-DLPFC, wernicke's area R-pSAC^2^ and L-pSAC^2^	10 hz rTMS +ct^5^	90%−110% RMT	1,200–1,400 x 30	6w	Sham coil
Yao et al. (22)	15/12	63.9/67.6	7/6	19.9/18.4	Moderate	Bilateral cerebellum crus ii	5 hz rTMS	90%RMT	2,000 x 2 x 20	4 w	Sham coil
Li et al. (30)	37/38	66.0/64.6	17/14	16.1/16.0	Moderate	Left DLPFC	20 hz rTMS	100%RMT	2,000 x 30	6 w	Sham coil
Roque et al. (42)	12/12	66.1/67.2	9/5	28.5/28.6	Mild	Left DLPFC	5 hz rTMS +ct^5^	100%RMT	1,500 x 30	10 w	Sham coil
Yan et al. (37)	35/34	71.4/73.4	25/23	15.7/15.6	Moderate	Left inferior parietal lobule	10 hz rTMS	100–110% RMT	800 x 10	2 w	Rotated 45°
Yuan et al. (38)	12/12	65.1/64.7	6/7	unclear	Unclear	Left DLPFC	10 hz rTMS	80%RMT	400 x 20	2 w	Tilted 90°
Sabbagh et al. (29)	79/50	76.9/76.7	38/21	21.7/21.3	21.7/21.3	Broca's area; Wernicke's area; left and right DLPFC; left and right IPL^3^	10 hz rTMS	110%RMT	1,300 × 30	6 w	Sham coil
Bagattini et al. (31)	27/23	73.6/73.4	10/11	23.7/22.8	23.7/22.8	Left DLPFC	20 hz rTMS +CT^5^	100%RMT	2,000 x 20	4 w	Thick wood block
Padala et al. (32)	9/11	74.3/79.6	1/1	22.9/21.4	Mild	Left DLPFC	10 hz rTMS	120%RMT	3,000 x 20	4w	Sham coil
Brem et al. (39)	16/10	69.3/69.1	12/5	21.2/22.0	21.2/22.0	Wernicke area,Broca's area, left and right DLPFC; left and right IPL^3^	10 hz rTMS +CT^5^	120%RMT	900 x 30	6 w	Sham coil
Zhang et al. (34)	15/13	69.0/68.5	12/10	20.5/19.8	Moderate	Left DLPFC and the left LTL^4^	10 hz rTMS +CT^5^	100%RMT	1,000 x 2 x 20	4 w	Conditional coil
Padala et al. (43)	8/8	65.6/65.6	0/0	25.6/25.6	25.6/25.6	Left DLPFC	10 hz rTMS	120%RMT	3,000 x 10	2 w	Sham coil
Koch et al. (44)	14/14	70.0/70.0	7/7	26.1/26.1	Mild	Bilateral precuneus	20 hz rTMS	100%RMT	1600 x 10	2 w	Sham coil
Zhao et al. (27)	17/13	69.3/71.4	10/7	22.2/22.8	Mild and moderate	Parietal and posterior temporal	20 hz rTMS +CT^5^	Unclear	2,400 x 30	6 w	Same sounds
Marra et al. (33)	15/19	65.1/65.2	9/13	24.5/24.2	Mild	Left DLPFC^1^	10 hz rTMS	110%RMT	2,000 x 10	2 w	Sham coil
Rabey et al. (41)	7/8	72.6/75.4	2/3	22.0/22.0	Mild	Left and right DLPFC,broca and wernicke, left and right pSAC^2^	10 hz rTMS +CT^5^	90–110% RMT	1300 x 30	6 w	Sham coil
Ahmed et al. 20 hz (28)	15/15	65.9/68.3	10/10	14.7/13.9	Moderate and severe	Left and right DLPFC	20 hz rTMS	90%RMT	4,000 x 5	1 w	Elevated coil
Ahmed et al. 1 hz(28)	15/15	68.6/68.3	9/10	12.7/13.9	Moderate and severe	Left and right DLPFC	1 hz rTMS	100%RMT	4,000x5	1 w	Elevated coil
Cotelli et al. (40)	5/5	71.2/74.4	unclear	16.2/16.0	Moderate	Left DLPFC	20 hz rTMS	100%RMT	2,000 x 20	4 w	A placebo coil

^1^DLPFC, dorsolateral performance cortex; ^2^pSAC, parietal somato-sensory association cortex; ^3^IPL, inferior parietal lobule; ^4^LTL, left lateral temporal lobe; ^5^CT, cognitive training; ^6^MMSE, Mini Mental Status Examination.

### Quality assessment of the studies

Table 2 shows that all the included studies had similar active and sham groups, and three-quarters of the studies described specific random allocation methods. Seven studies employed a triple-blind design, whereas four studies utilized a double-blind design. The dropout number of patients was reported in 18 studies. Studies with a high potential risk of bias (scores < 4) were removed for sensitivity analysis.

**Table 2:** The quality assessment of the included studies.

	**Accepted standardized criteria used for AD/MCI**	**Specific random sequence generation methods**	**Blinding of personnel and participants**	**Blinding of outcome assessment**	**Similar characteristics in active and sham groups**	**Reports of drop-out number of the participants**	**Overall risks**
Wei et al. (45)	1	1	0	1	1	1	5
Koch et al. (44)	1	0	1	1	1	1	5
Wu et al. (35)	1	1	0	un	1	1	4
Vecchio et al. (36)	1	un	un	un	1	1	3
Yao et al. (22)	1	1	1	0	1	un	4
Li et al. (30)	1	1	un	1	1	1	5
Roque et al. (44)	1	1	1	un	1	1	5
Yan et al. (37)	1	1	0	1	1	1	5
Yuan et al. (38)	1	1	0	1	1	1	5
Sabbagh et al. (29)	1	1	1	1	1	1	6
Bagattini et al. (31)	1	un	1	1	1	1	5
Padala et al. (32)	1	1	1	1	1	1	6
Brem et al. (39)	1	0	1	un	1	1	4
Zhang et al. (20)	1	1	0	1	1	1	5
Padala et al. (43)	1	1	1	1	1	1	6
Koch et al. (46)	1	0	un	1	1	1	4
Zhao et al. (27)	1	0	0	1	1	0	3
Marra et al. (44)	1	1	1	1	1	1	6
Rabey et al. (41)	1	1	0	un	1	1	4
Ahmed et al. (28) 20 hz	1	1	1	1	1	un	5
Ahmed et al. (28) 1 hz	1	1	1	1	0	un	4
Cotelli et al. (40)	1	0	un	1	1	1	4

^1^means compliance with the assessment; ^0^means non-compliance with the assessment; ^un^means not reported.

### Effects of TMS on AD

Given that most of the included studies reported short-term results (< 3 days) as well as follow-up (>4 weeks) outcomes, the pooled effects of TMS on the cognitive improvement in MCI and AD patients were assessed for both outcome timings.

**Figure 2:**
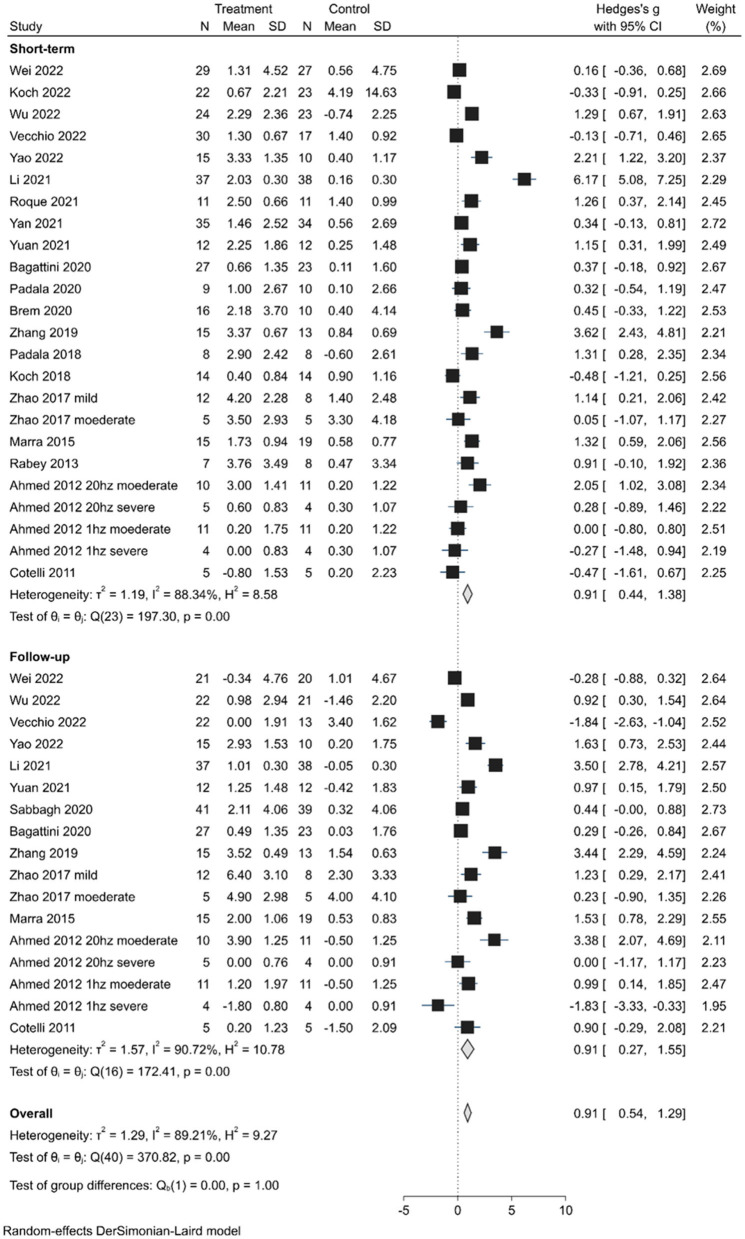
Forest plot depicting the pooled short-term and long-lasting effects of TMS on cognition outcomes.

The short-term effects were evaluated in 24 trials (20 studies) and demonstrated an overall significant improvement (SMD, 0.91; 95% CI 0.44–1.38; *P* < 0.01) in the random-effects model analysis. Of the 24 trials, 10 showed statistically significant positive effects (Figure 2). Long-lasting effects (SMD, 0.91; 95% CI 0.27–1.55; *P* < 0.01) were observed in 17 trials (13 studies), with significant positive effects in 9 trials and negative effects in 2 trials. However, the difference in the effects between the short-term and long-lasting outcomes was not statistically significant (Q = 0.00, *P* < 1.00). Substantial heterogeneity was found in both short-term (H^2^ = 8.58, I^2^ = 88.34%) and long-lasting effects (H^2^ = 9.19, I^2^ = 89.21%). Therefore, subgroup analyses were performed explore the potential sources of heterogeneity and assess the efficacy of various factors on cognitive improvement.

### Subgroup analyses

**Figure 3:**
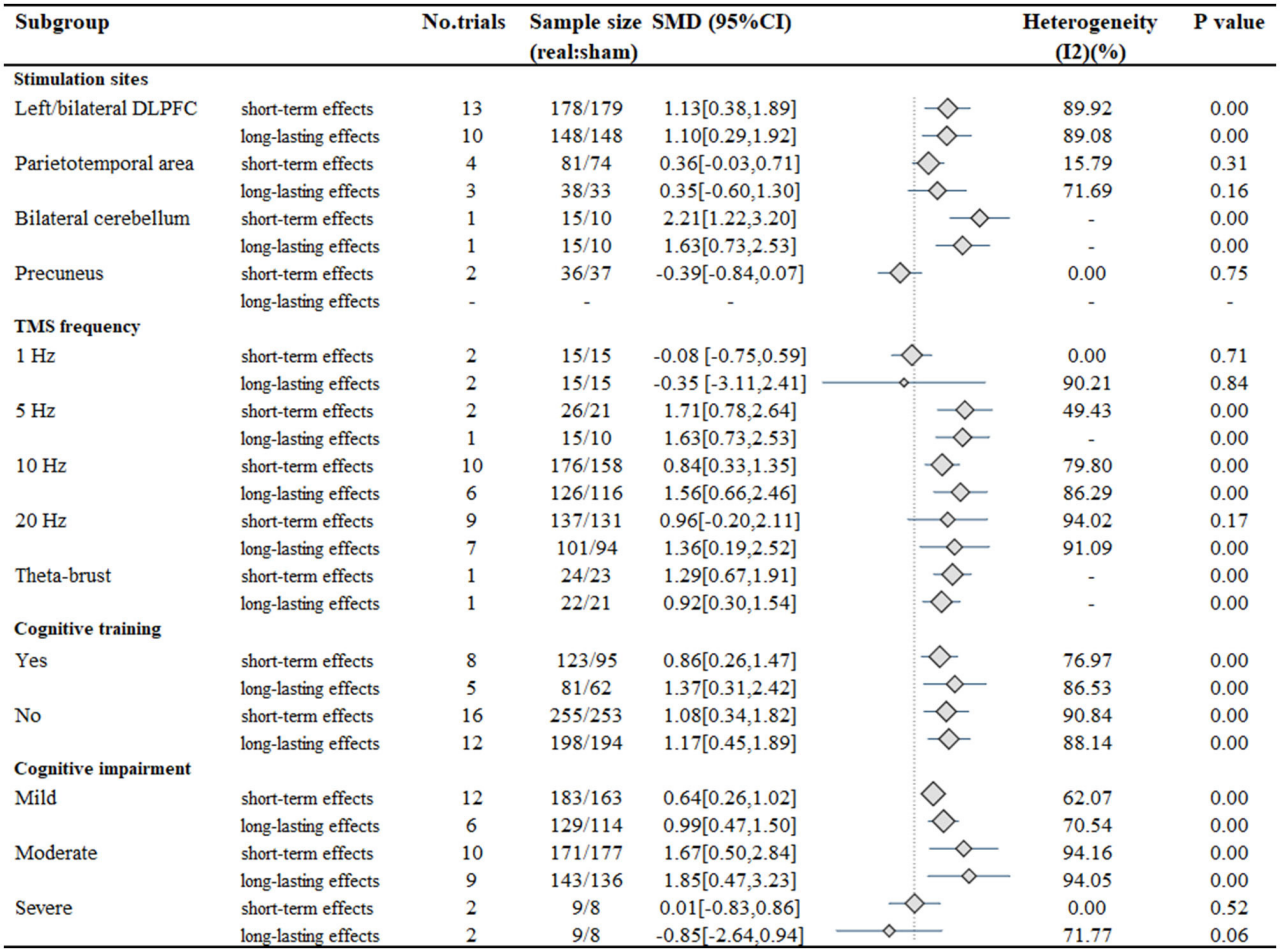
Results of the subgroup analysis.

Figure 3 shows the subgroup analysis of the stimulation sites. The results indicate that global cognitive function could be significantly improved in the short- and long-lasting terms with left/bilateral DLPFC stimulation (short-term effects: SMD, 1.13; 95% CI 0.38–1.89; *P* < 0.01; long-lasting effects: SMD, 1.10; 95% CI 0.29–1.92; *P* < 0.01) and bilateral cerebellum stimulation (short-term effects: SMD, 2.21; 95% CI 1.22–3.20; *P* < 0.01; long-lasting effects: SMD, 1.63; 95% CI 0.73–2.53; *P* < 0.01). However, parietotemporal area stimulation (short-term effects: SMD, 0.36; 95% CI −0.03–0.71; *P* = 0.31; long-lasting effects: SMD, 0.35; 95% CI −0.60–1.30; *P* = 0.16) and precuneus stimulation (short-term effects: SMD, −0.39; 95% CI −0.84–0.07; *P* = 0.75) did not show significant improvement. Subgroup analyses comparing multiple site and bilateral site stimulation with single-site stimulation did not demonstrate larger responses (short-term effects: Test of group differences: Q = 1.02, *P* = 0.60; long-lasting effects: Test of group differences: Q = 0.41, *P* = 0.82) (Supplementary Figures 1, 2). Furthermore, stimulation of the left DLPFC alone, which showed the largest effect size (short-term effects: SMD, 1.39; 95% CI 0.43–2.36; long-lasting effects: SMD, 1.35; 95% CI 0.39–2.32), showed no difference (short-term effects: Test of group differences: Q = 3.61, *P* = 0.16; long-lasting effects: Test of group differences: Q = 0.45, *P* = 0.80) with bilateral DLPFC stimulation and multiple site stimulation involving the left/bilateral DLPFC (Supplementary Figures 3, 4). Additionally, although stimulation of the bilateral cerebellum showed larger responses (short-term effects: SMD, 2.21; 95% CI 1.22–3.20; long-lasting effects: SMD, 1.39; 95% CI 0.55–2.23) than left DLPFC stimulation, the difference was not statistically significant (short-term effects: Test of group differences: Q = 1.34, *P* = 0.25; long-lasting effects: Test of group differences: Q = 0.17, *P* = 0.68) (Supplementary Figures 5, 6).

The stimulation frequency of rTMS ranged from 1 Hz to 20 Hz, in addition to a study that applied 50 Hz intermittent theta-burst stimulation (iTBS). Subgroup analysis of stimulation frequency revealed that cognitive outcomes were improved in the short-term responses in patients receiving 5 Hz stimulation (SMD, 1.71; 95% CI 0.78–2.64; *P* < 0.01), 10 Hz stimulation (SMD, 0.84; 95% CI 0.33–1.35; *P* < 0.01) and iTBS (SMD, 1.29; 95% CI 0.67–1.91; *P* < 0.01), but not in 1 Hz stimulation (SMD, −0.08; 95% CI −0.75–0.59; *P* = 0.71) or 20 Hz stimulation (SMD, 0.96; 95% CI −0.20–2.11; *P* = 0.17). In terms of long-lasting effects, all stimulation frequencies achieved statistical significance (5 Hz: SMD, 1.63; 95% CI 0.73–2.53; *P* < 0.01; 10 Hz: SMD, 1.56; 95% CI 0.66–2.46; *P* < 0.01; 20 Hz: SMD, 1.36; 95% CI 0.19–2.52; *P* < 0.01; iTBS: SMD, 0.92; 95% CI 0.30–1.54; *P* < 0.01) expect for 1 Hz stimulation (SMD, −0.35; 95% CI −3.11–2.41; *P* = 0.84).

Furthermore, significant improvements in cognitive function were observed with TMS combined with CT (short-term effects: SMD, 0.86; 95% CI 0.26–1.47; *P* < 0.01; long-lasting effects: SMD, 1.08; 95% CI 0.34–1.82; *P* < 0.01) or without CT (short-term effects: SMD, 1.37; 95% CI 0.31–2.42; *P* < 0.01; long-lasting effects: SMD, 1.17; 95% CI 0.45–1.89; *P* < 0.01). The TMS combination with CT showed no significant difference with TMS without CT (short-term effects: Test of group differences: Q = 0.01, *P* = 0.91; long-lasting effects: Test of group differences: Q = 0.26, *P* = 0.61). Subgroup analysis of CT vs. no CT in MCI patients also did not reveal a significant difference between the groups (short-term effects: Test of group differences: Q = 0.09, *P* = 0.76; long-lasting effects: Test of group differences: Q = 0.07, *P* = 0.80) (Supplementary Figures 7, 8). Additionally, in the subgroup analysis of patient characteristics, patients with mild cognitive impairment (short-term effects: SMD, 0.64; 95% CI 0.26–1.02; *P* < 0.01; long-lasting effects: SMD, 0.99; 95% CI 0.47–1.50; *P* < 0.01) or moderate cognitive impairment (short-term effects: SMD, 1.67; 95% CI 0.50–2.84; *P* < 0.01; long-lasting effects: SMD, 1.85; 95% CI 0.47–3.23; *P* < 0.01) showed greater improvement than participants with severe cognitive impairment (short-term effects: SMD, 0.01; 95% CI −0.83–0.86; *P* = 0.52; long-lasting effects: SMD, −0.85; 95% CI −2.64–0.4; *P* = 0.06).

### Publication bias and sensitivity analysis

**Figure 4:**
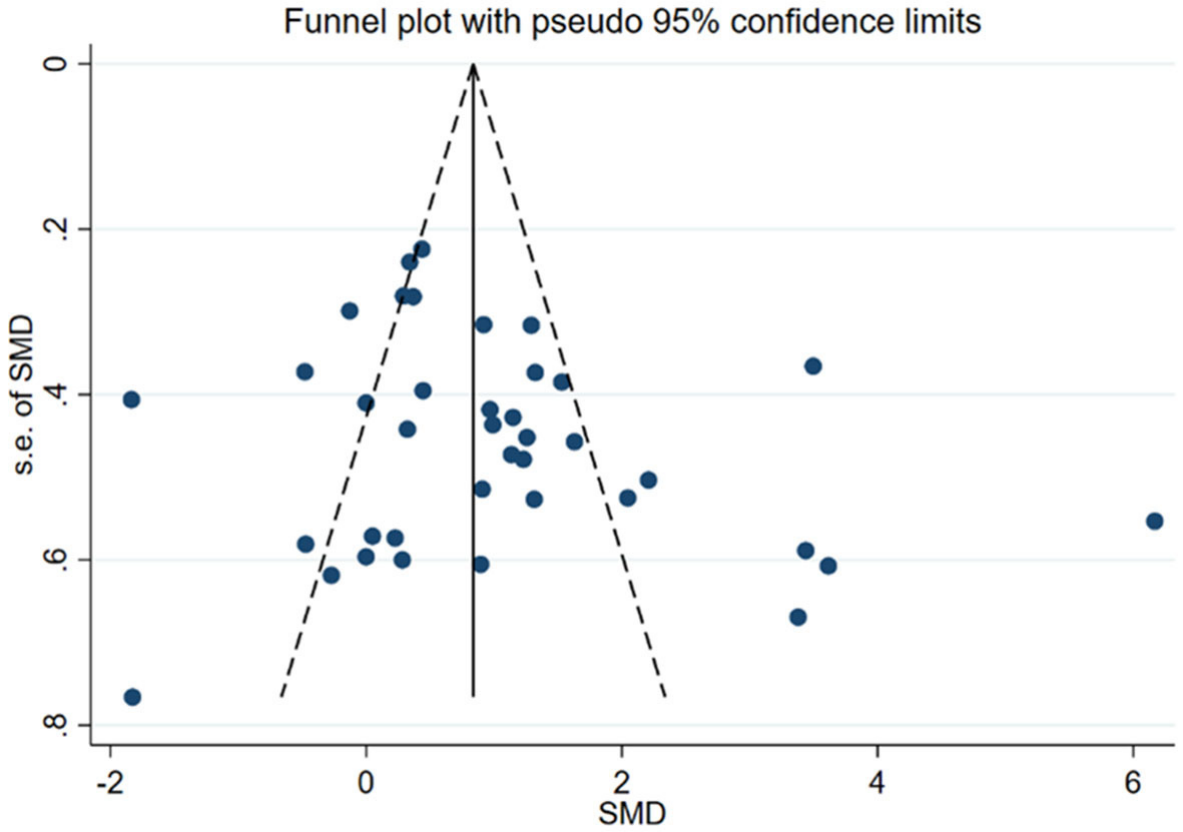
Funnel plot illustrating potential publication bias.

**Figure 5:**
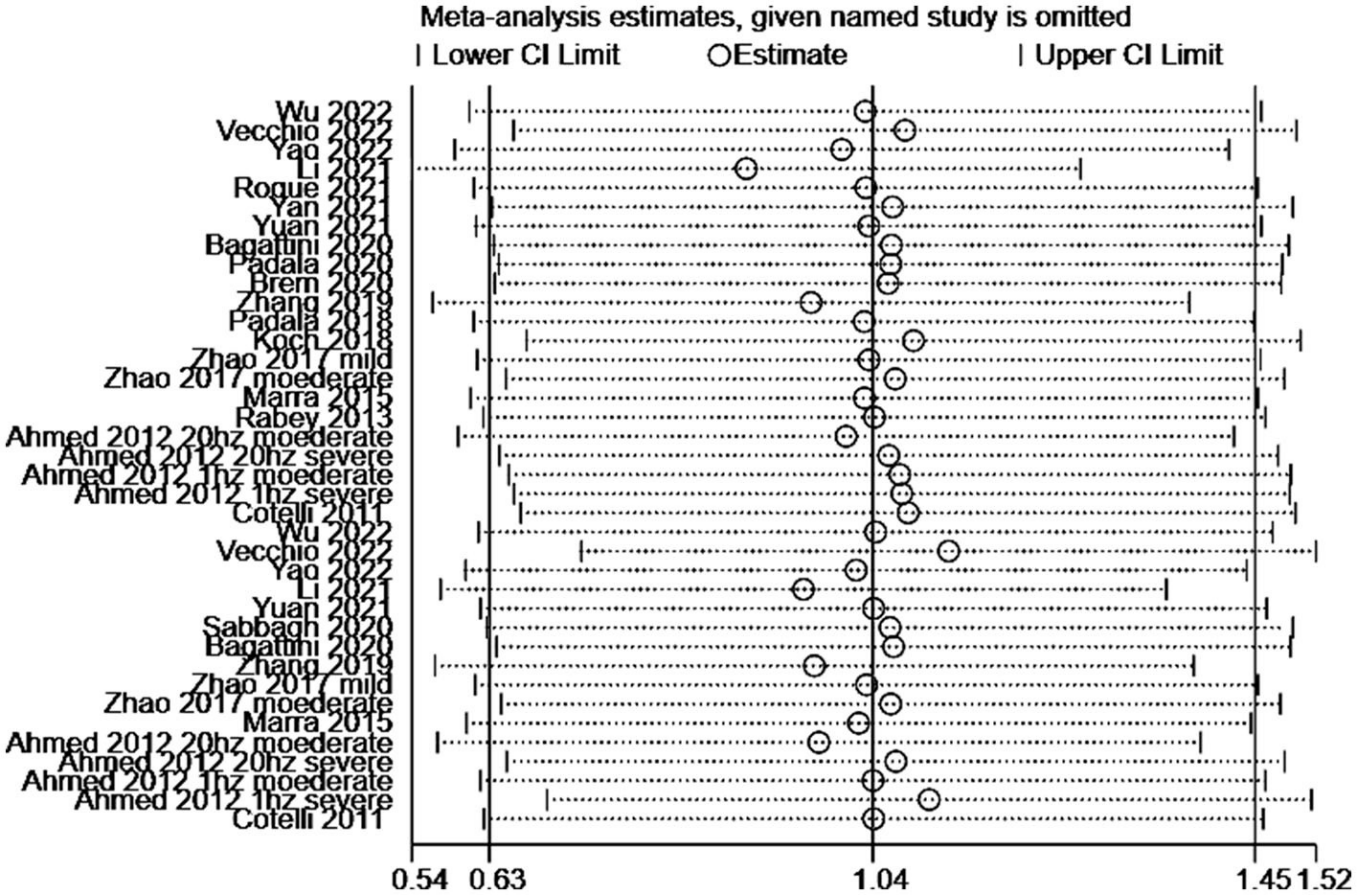
Sensitivity analysis examining the robustness of the results.

Figure 4, displays the results of Egger's test (*P* = 0.115) and visual inspection of the funnel plot, which did not indicate any significant publication bias in the primary outcome. Therefore, no fill-and-trim procedure was performed to adjust the effect sizes. Additionally, sensitivity analysis was conducted to examine the impact of each included study on the overall results. Figure 5 illustrates the results of omitting each study. It can be observed that the exclusion of any specific study did not lead to substantial changes in the overall findings, indicating the robustness and reliability of our results.

## Discussion

This meta-analysis revealed the following: (1) Stimulation of the DLPFC and cerebellum had short-term and long-lasting positive effects on general cognitive function. (2) TMS protocols using moderate frequency stimulation (5 Hz and 10 Hz) and iTBS demonstrated larger therapeutic effects. (3) CT did not yield any additional effects. (4) The population with mild to moderate cognitive impairment responded better to stimulation than patients with severe AD.

Our study is the first to demonstrate that the cerebellum, in addition to the DLPFC, is an effective TMS site for AD treatment. Previous studies predominantly focused on bilateral or left DLPFC stimulation as the TMS site (28, 30–33, 35, 38, 40, 42, 43). Other reported stimulation sites, such as the parietotemporal area (27, 37) and the precuneus (44) showed limited improvement in global cognition. Existing meta-analyses on TMS efficacy in AD treatment primarily compared single and multiple stimulation sites (16, 48, 49) or left, right, and bilateral hemisphere stimulation (17, 50). Our subgroup analyses revealed no significant difference between 10 trials using single-site stimulation and 6 trials using bilateral site stimulation or 6 trials using multiple site stimulation. Jiang et al. (17) reported positive effects of left/bilateral DLPFC stimulation. Our comparisons of the left DLPFC, bilateral DLPFC, and multiple site stimulation (including the left/bilateral DLPFC) indicated that the left DLPFC showed the largest improvement. However, bilateral cerebellum stimulation had a larger effect size than left DLPFC stimulation, although the difference was not statistically significant. Subgroup analyses of four trials using parietotemporal stimulation and two trials using precuneus stimulation showed no significant therapeutic effects. Thus, among all reported stimulation sites, the left DLPFC and cerebellum stimulation were more effective. Although only one study (22) applied bilateral cerebellum stimulation, the potential of cerebellar stimulation in AD treatment deserves more attention, and further investigation is needed in future studies.

Regarding stimulation frequency, our subgroup analyses indicated that moderate frequency stimulation (5 Hz and 10 Hz) and theta-burst stimulation might have superior efficacy for general cognition compared to low-frequency (1 Hz) and high-frequency (20 Hz) stimulation. Among these frequencies, the 5 Hz stimulation demonstrated the highest effect size for both short-term and long-lasting effects. The 20 Hz stimulation was only significant for the long-lasting effects, and its effect size was lower than that of the 10 Hz stimulation. Theta-burst stimulation exhibited high short-term effects but the lowest long-lasting effects. Generally, low-frequency ( ≤ 1 Hz) is considered to suppres excitability within the targeted brain region, while higher frequency stimulation is considered enhancing, but the inhibitory or excitatory effects on brain areas do not directly correspond to a decrease or improvement in cognitive function. The improved performance may be observed with an optimum frequency depending on the task demands and stimulation area (51). Most studies considered 5, 10, and 20 Hz as high-frequency stimulation and concluded that high-frequency stimulation has excitatory effects (12, 17, 21, 50, 52). Wang et al. (15) considered 10 Hz as a moderate frequency and 20 Hz as a high-frequency and their findings contradicted our research, suggesting that 20 Hz stimulation induced better cognitive improvement than 10 Hz rTMS. Several studies have also reported blockade or disruption of brain function with high-frequency TMS over certain brain regions (53, 54). Regarding the duration of treatment required for effectiveness, all frequencies of rTMS demonstrated positive significance for long-lasting effects, except for 1 Hz, probably due to the short-lived block effects of high-frequency TMS. Thus, this meta-analysis is the first to demonstrate that moderate-frequency (5 Hz and 10 Hz) rTMS may be more suitable than high- and low- frequency rTMS for AD treatment.

The combination of CT with multisite rTMS has shown potential effectiveness in enhancing cognition (55). However, our findings did not provide evidence for additional cognitive enhancement through CT, independent of TMS. Previous meta-analyses have reported both positive (14) and not positive additional effects (12, 16). Wang et al. proposed that the positive effects of combining CT can be confounded by the number of stimulation sessions (14). Chu et al. (12) also observed a lack of efficacy when CT was combined with TMS in different cognitive domains. Considering that CT may be more effective in the early stages of AD, we conducted a subgroup analysis comparing rTMS combined with CT and rTMS alone in individuals with mild cognitive impairment (MCI) and mild AD. No significant differences were observed between the two subgroups. However, it is important to note that the effect of CT cannot be deemed null because the recovery effect of CT may not have been sufficient to induce a significant additive effect in conjunction with TMS.

Regarding participant characteristics, our subgroup analysis revealed that patients with severe AD may not experience as much improvement as individuals with mild-to-moderate AD through TMS, which is consistent with previous meta-analyses (12, 16, 21).

As research on TMS progress, novel methodologies have emerged (22) and previous studies have been enriched, leading to new changes in the analysis of TMS treatment effects on MCI and AD patients. The inclusion of recently published RCTs comprehensive comparisons of factors influencing TMS efficacy, have resulted in different conclusions compared with previous studies. This meta-analysis is the first meta-analysis to demonstrate that the cerebellum may be a potent TMS site for improving cognitive function, whereas previous studies lacked convincing evidence for cognition enhancement through TMS targets other than the DLPFC (55). Additionally, instead of generalizing stimulation higher than 1 Hz as high-frequency stimulation, we investigated the effect size of 5 Hz, 10 Hz, 20 Hz stimulation and proposed that moderate frequency (5 Hz and 10 Hz) rTMS may be more effective for general cognition than high-frequency (20 Hz) rTMS. Comprehensive comparisons conducted through four subgroup analyses are helpful in identifying the optimal stimulation protocols and appropriate patient characteristics. Moreover, our analysis included 21 TMS RCTs based on rigorous inclusion criteria, enriching existing studies and providing a higher level of evidence in this field. The absence of significant publication bias and consistent results in the sensitivity analysis further validate the reliability of our findings. In summary, our study demonstrates that the cerebellum is a potential novel TMS target for improving cognition function, in addition to the DLPFC, and suggests that 5 Hz and 10 Hz stimulation may be more effective than 20 Hz rTMS. Future studies should focus on identifying the optimal combination of TMS parameters, including the stimulation site and frequency, as well as tailoring TMS treatment protocols to individual patients.

However, it is important to acknowledge several limitations of this study. First, although 21 studies and 25 trials were included, the sample size was relatively small and uneven, highlighting the need for further RCTs to provide more robust evidence. Second, our meta-analysis focused only on global cognitive outcomes, whereas other domains, such as behavioral and psychological changes associated with cognitive impairment, require further research. Third, the inclusion of both MCI and AD patients, representing distinct clinical stages of cognitive impairment, along with the heterogeneity of the study subjects, was intended to increase the statistical reliability and broaden the general applicability. However, studies specifically focusing on patients with MCI or pure AD may yield less interference. Finally, certain findings were obtained from a single study with a small sample size, necessitating confirmation through large-scale clinical trials.

## Conclusion

Our data suggested that stimulation of the left DLPFC or bilateral cerebellum, along with moderate frequency stimulation (5 Hz and 10 Hz), may yield more favorable outcomes than other TMS protocols in terms of improving global cognition. Additionally, and patients with mild-to-moderate AD appear to have better responses to TMS than those with severe AD. However, the additional benefits of combining CT or multiple site stimulation lack sufficient supportive evidence. The cognitive improvement effects of TMS persisted at the 4-week follow-up.

## Data availability statement

The original contributions presented in the study are included in the article/Supplementary material, further inquiries can be directed to the corresponding author.

## Author contributions

YY: writing - original draft, methodology, investigation, formal analysis, and visualization. MT: validation, writing - original draft, and investigation. TW: investigation and resources. XW: data curation. YW: investigation. JS: conceptualization, funding acquisition, resources, supervision, writing - reviewing, and editing.

## Supplementary material

Supplementary Figure 1 Forest plot: the subgroup analysis of short-term effects of single site, bilateral site, and multiple site stimulation.

Supplementary Figure 2 Forest plot: the subgroup analysis of long-lasting effects of single site, bilateral site, and multiple site stimulation.

Supplementary Figure 3 Forest plot: the subgroup analysis of short-term effects of left DLPFC stimulation, bilateral DLPFC stimulation, and multiple sites including DLPFC stimulation.

Supplementary Figure 4 Forest plot: the subgroup analysis of long-lasting effects of left DLPFC stimulation, bilateral DLPFC stimulation, and multiple sites including DLPFC stimulation.

Supplementary Figure 5 Forest plot: the subgroup analysis of short-term effects of left DLPFC and bilateral cerebellum stimulation.

Supplementary Figure 6 Forest plot: the subgroup analysis of long-lasting effects of left DLPFC and bilateral cerebellum stimulation.

Supplementary Figure 7 Forest plot: the subgroup analysis of short-term effects on mild cognitive impairment patients with CT or without CT.

Supplementary Figure 8 Forest plot: the subgroup analysis of long-lasting effects on mild cognitive impairment patients with CT or without CT.

Supplementary Figure 9 Forest plot: the subgroup analysis of the stimulation sites in the short-term effects.

Supplementary Figure 10 Forest plot: the subgroup analysis of the stimulation sites in the long-lasting effects.

Supplementary Figure 11 Forest plot: the subgroup analysis of the TMS frequency in the short-term effects.

Supplementary Figure 12 Forest plot: the subgroup analysis of the TMS frequency in the long-lasting effects.

Supplementary Figure 13 Forest plot: the subgroup analysis of the with/without CT in the short-term effects.

Supplementary Figure 14 Forest plot: the subgroup analysis of the with/without CT in the long-lasting effects.

Supplementary Figure 15 Forest plot: the subgroup analysis of patient characteristics in the short-term effects **(Above)** and in the long-lasting effects **(Below)**.
