# Supplementary figures and images for: Transcranial magnetic stimulation effects on cognitive enhancement in mild cognitive impairment and Alzheimer's disease: a systematic review and meta-analysis
Source: Front Neurol. 2023 Jul 17;14:1209205. doi: 10.3389/fneur.2023.1209205 (PMC10389278; doi:10.3389/fneur.2023.1209205)

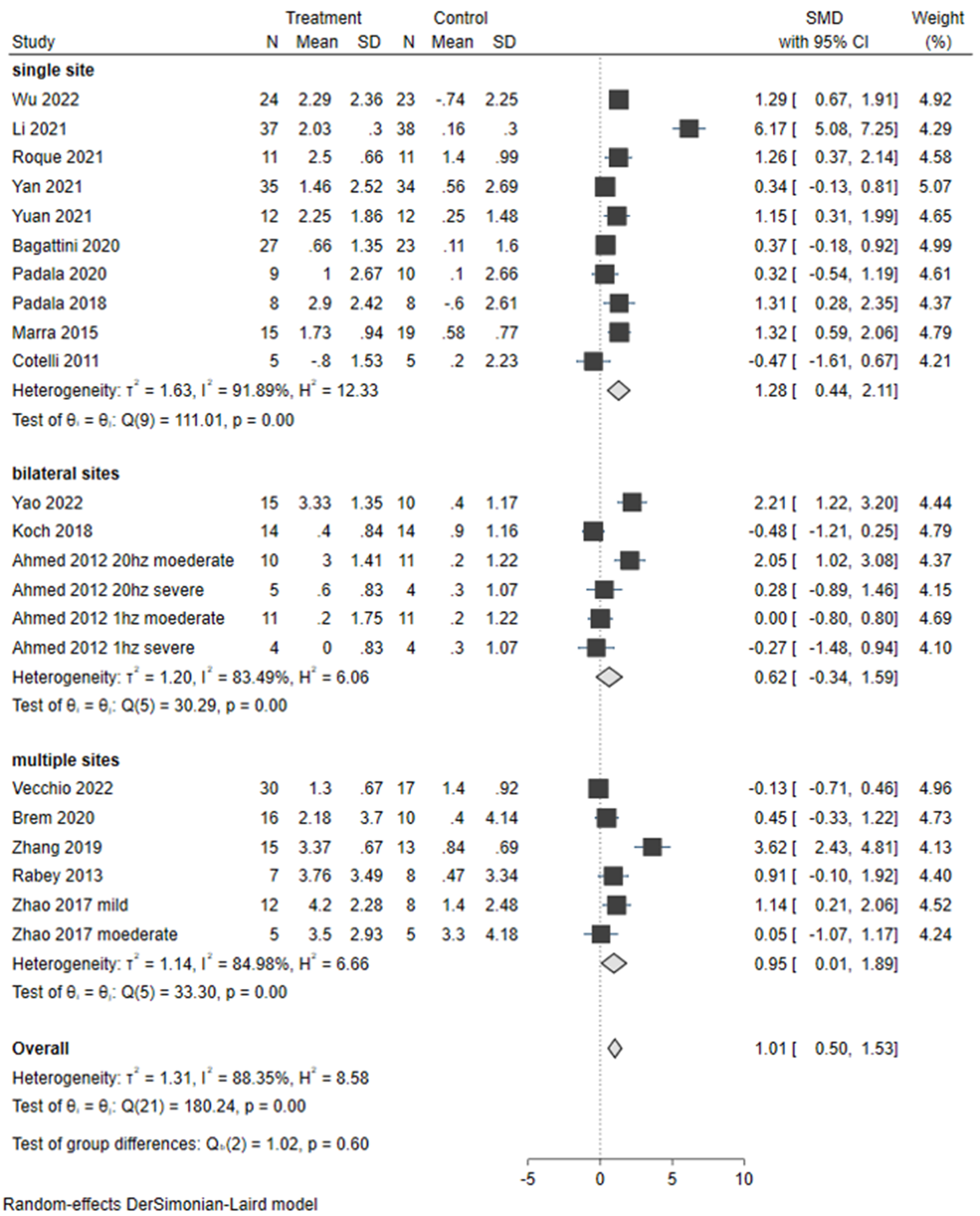

Supplement: Supplementary Figure 1 — Forest plot: the subgroup analysis of short-term effects of single site, bilateral site, and multiple site stimulation. [file Image_1.TIF]

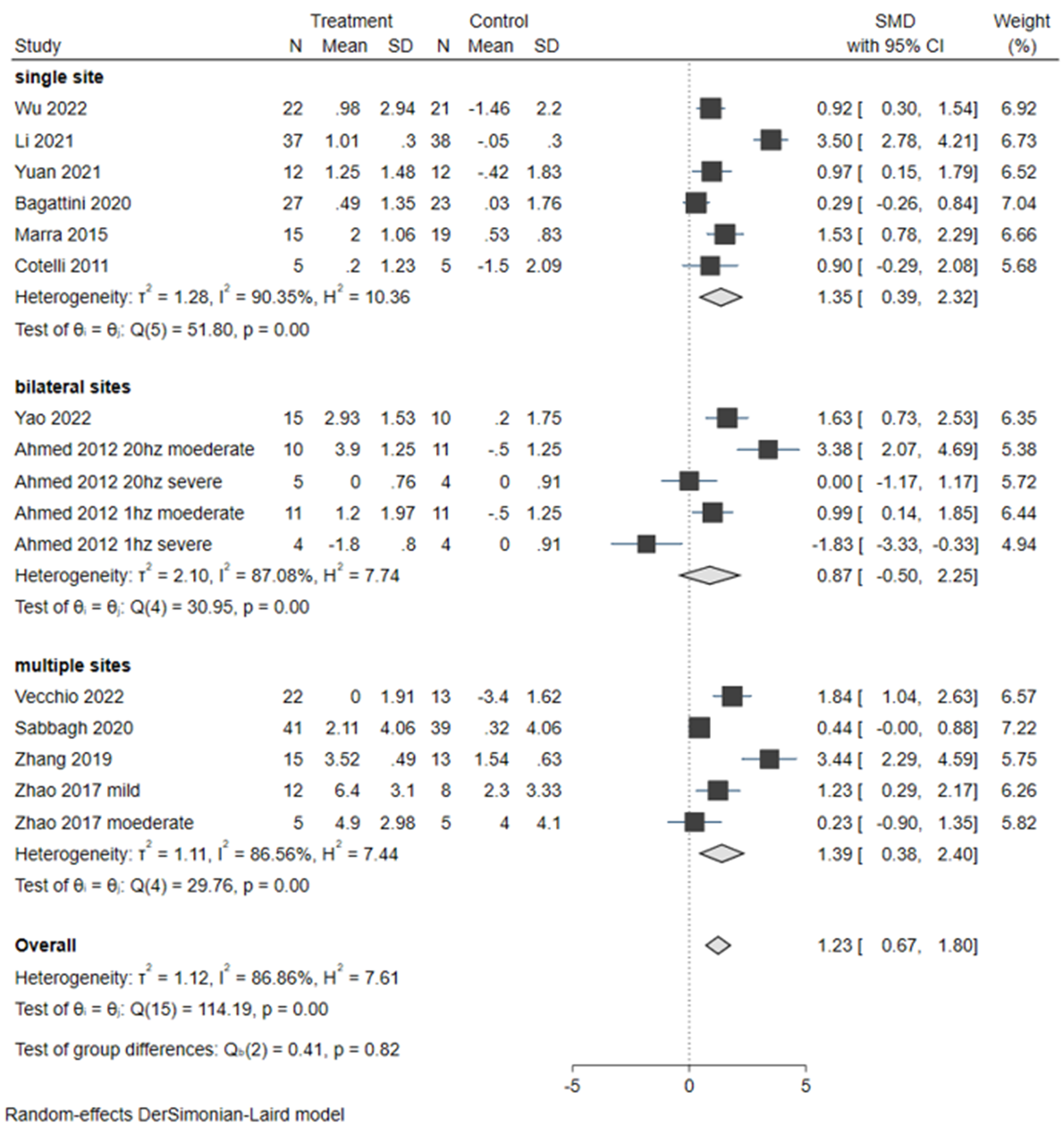

Supplement: Supplementary Figure 2 — Forest plot: the subgroup analysis of long-lasting effects of single site, bilateral site, and multiple site stimulation. [file Image_2.TIF]

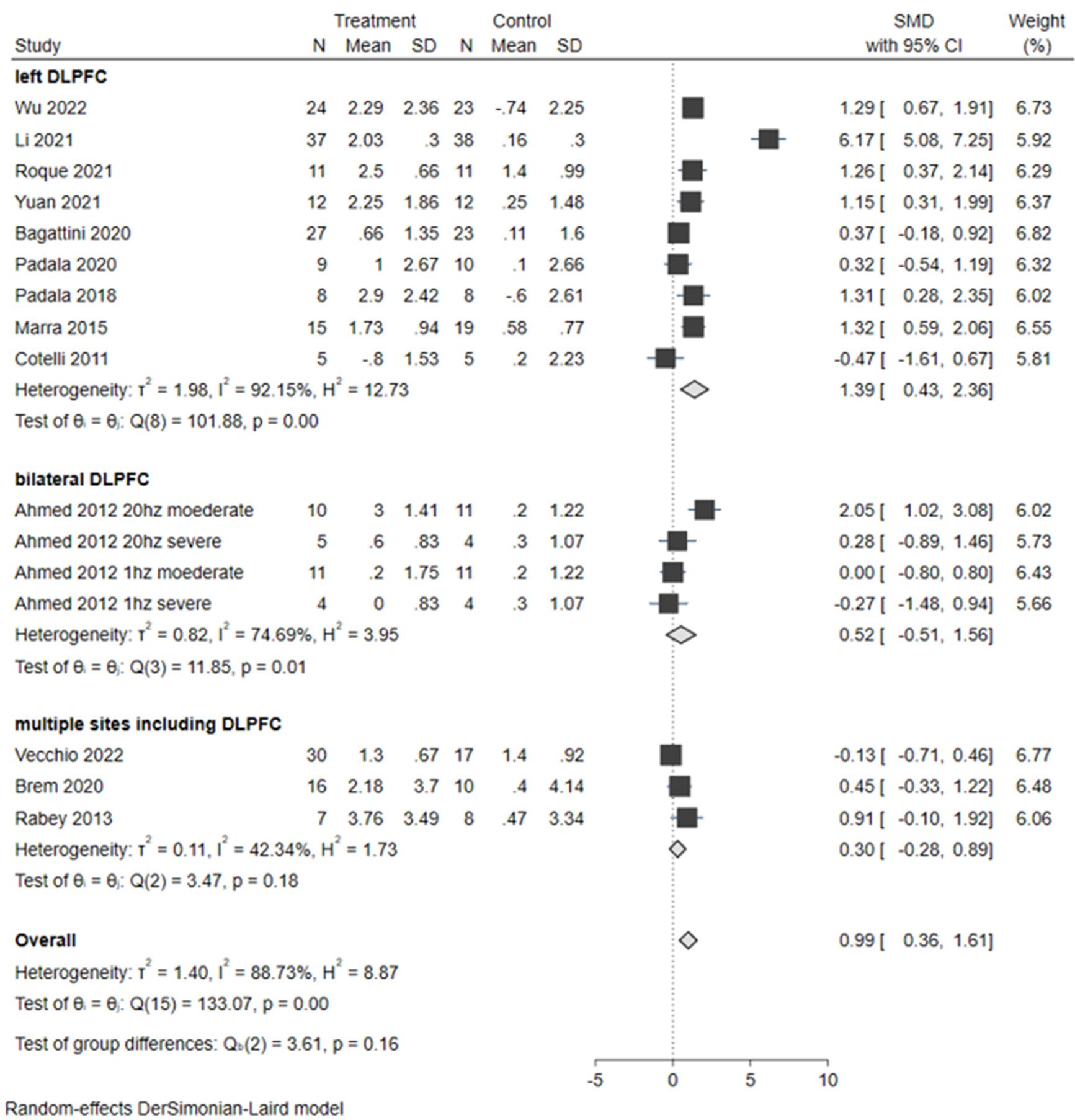

Supplement: Supplementary Figure 3 — Forest plot: the subgroup analysis of short-term effects of left DLPFC stimulation, bilateral DLPFC stimulation, and multiple sites including DLPFC stimulation. [file Image_3.TIF]

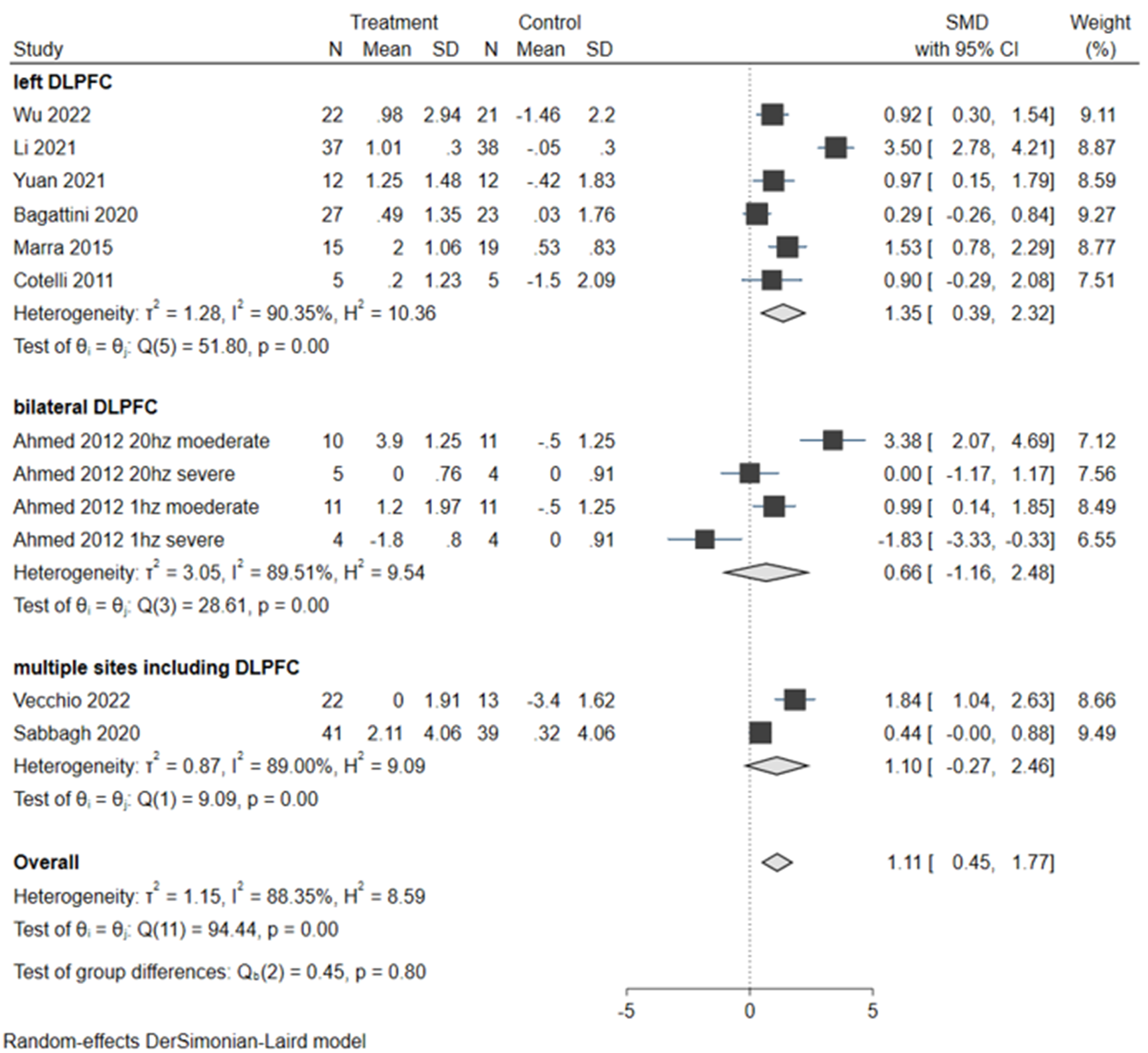

Supplement: Supplementary Figure 4 — Forest plot: the subgroup analysis of long-lasting effects of left DLPFC stimulation, bilateral DLPFC stimulation, and multiple sites including DLPFC stimulation. [file Image_4.TIF]

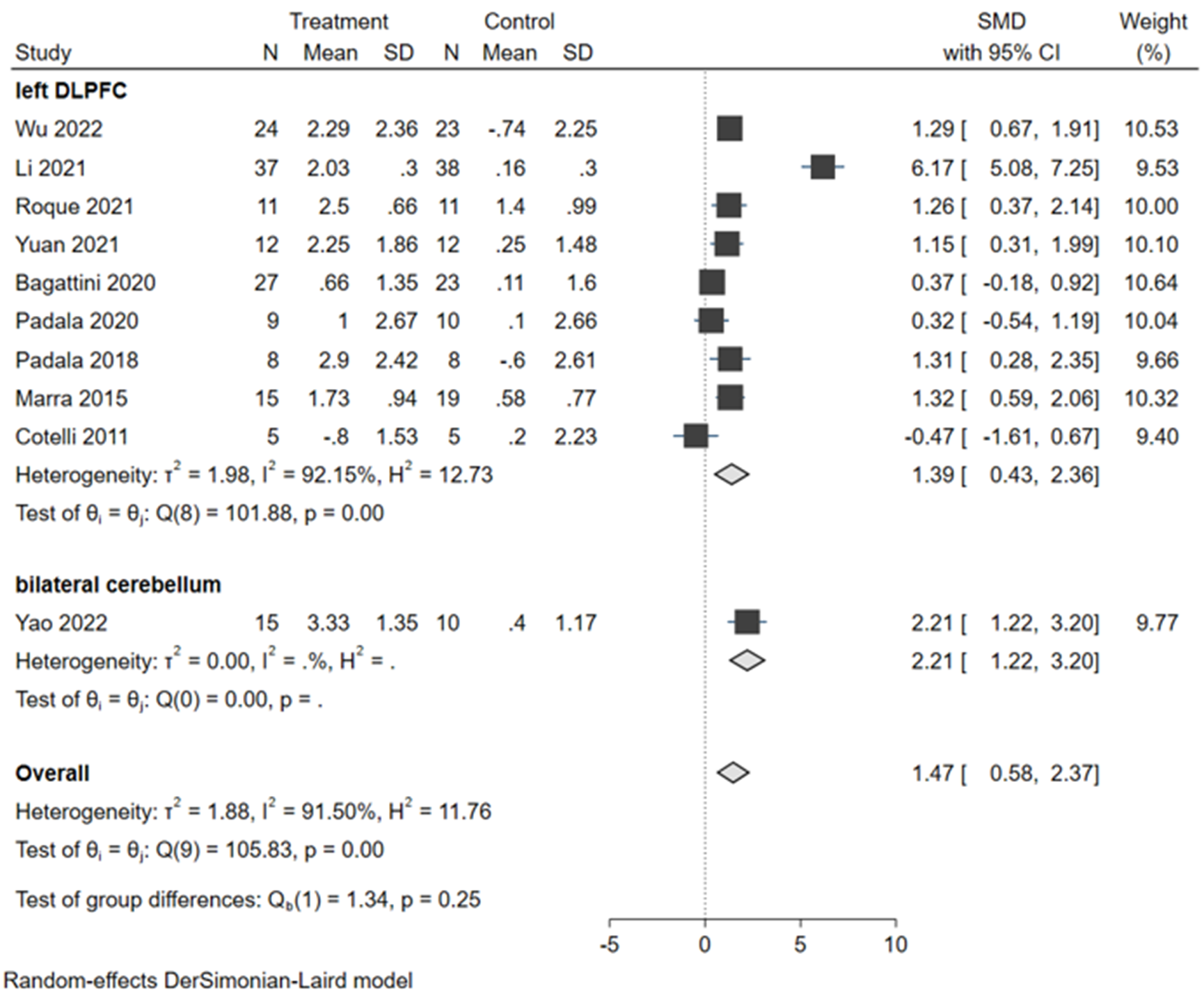

Supplement: Supplementary Figure 5 — Forest plot: the subgroup analysis of short-term effects of left DLPFC and bilateral cerebellum stimulation. [file Image_5.TIF]

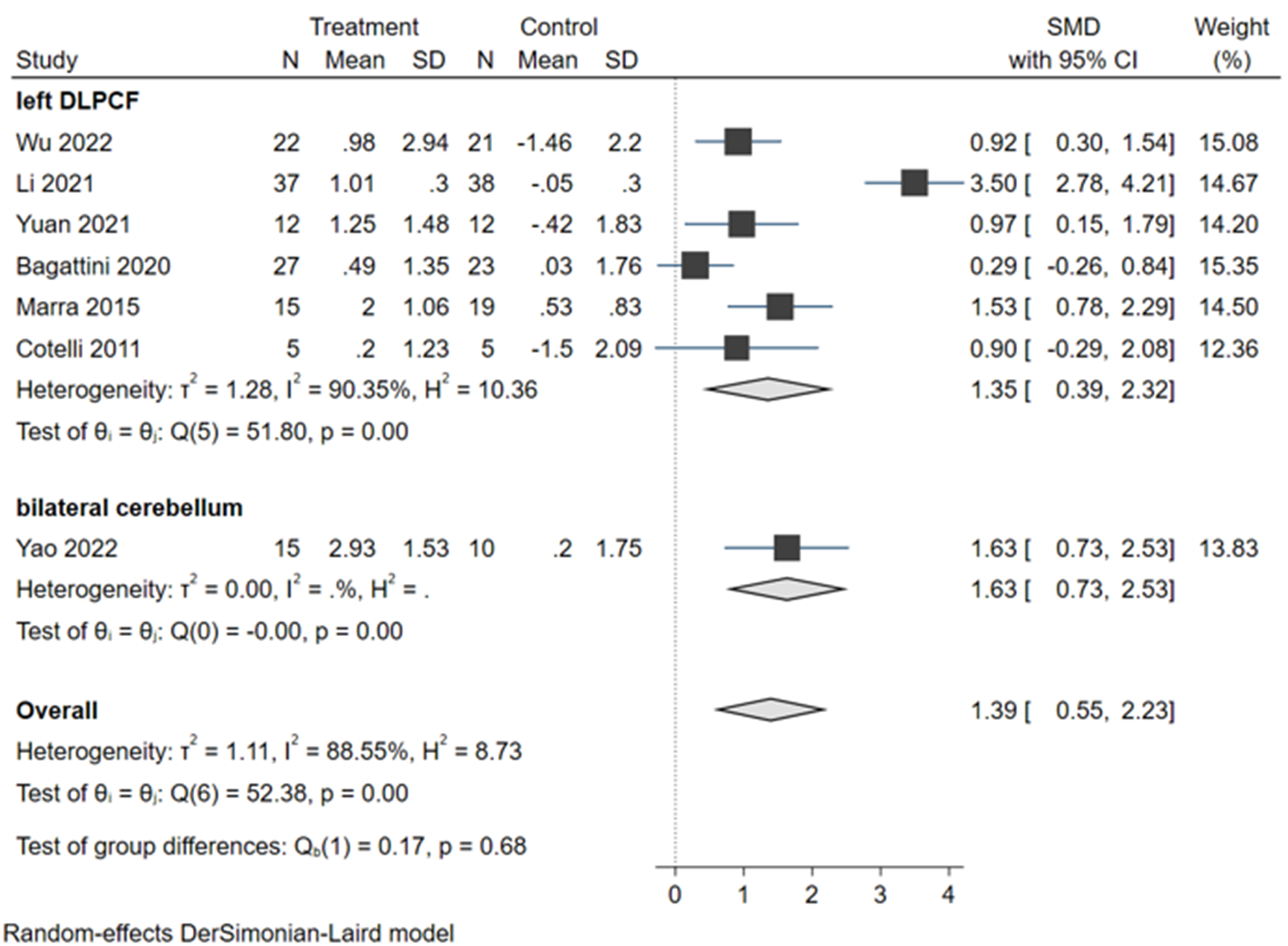

Supplement: Supplementary Figure 6 — Forest plot: the subgroup analysis of long-lasting effects of left DLPFC and bilateral cerebellum stimulation. [file Image_6.TIF]

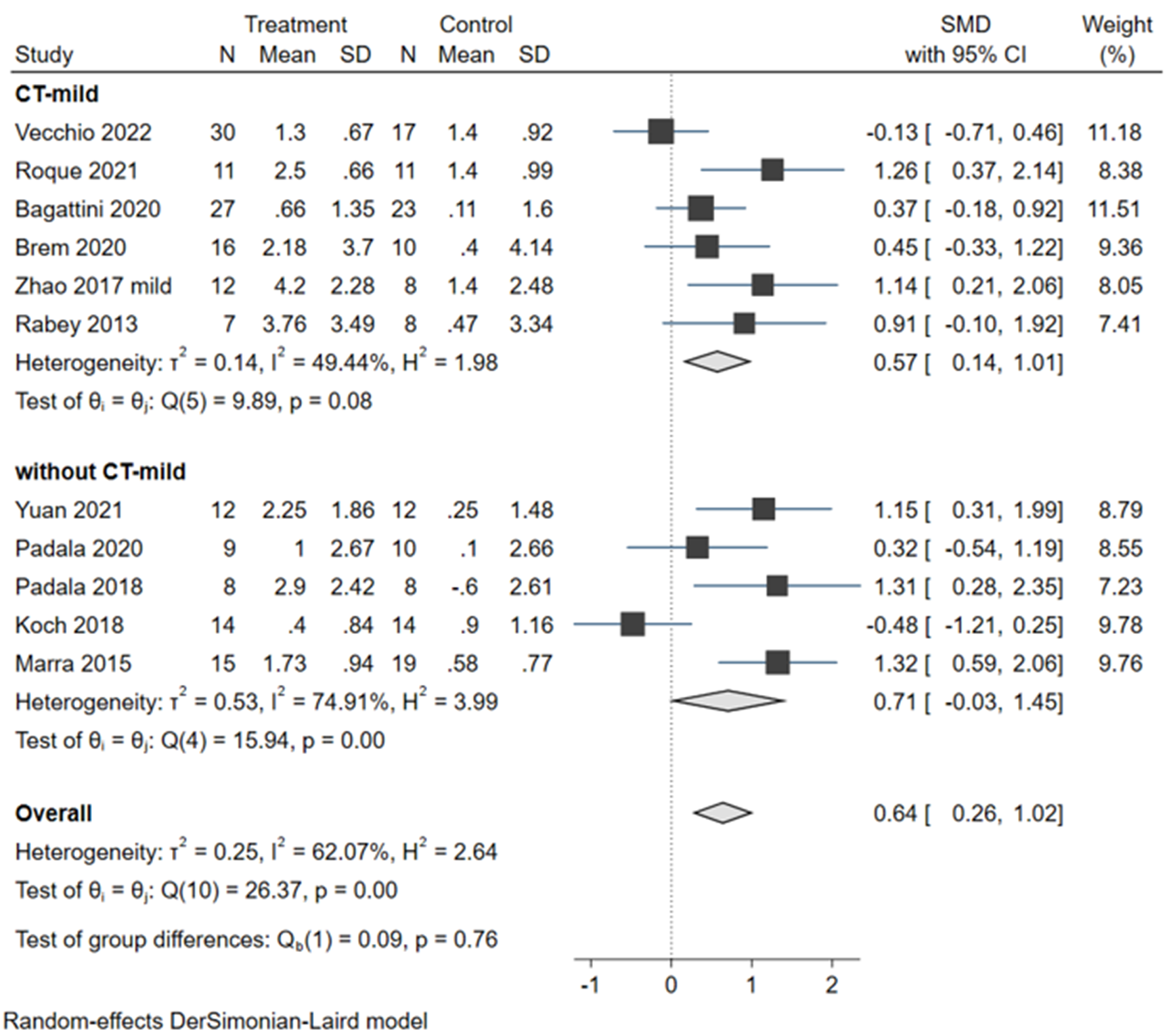

Supplement: Supplementary Figure 7 — Forest plot: the subgroup analysis of short-term effects on mild cognitive impairment patients with CT or without CT. [file Image_7.TIF]

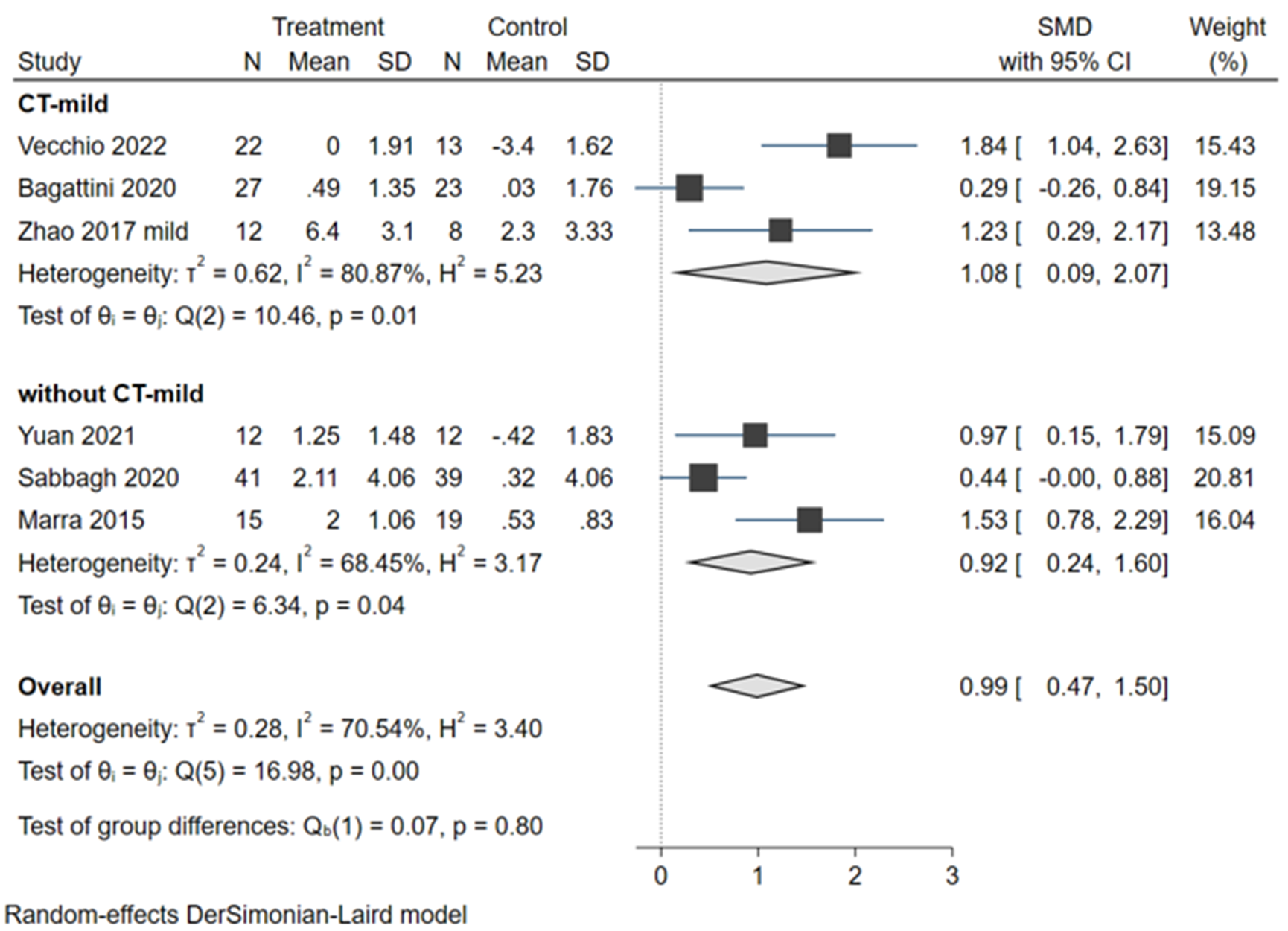

Supplement: Supplementary Figure 8 — Forest plot: the subgroup analysis of long-lasting effects on mild cognitive impairment patients with CT or without CT. [file Image_8.TIF]

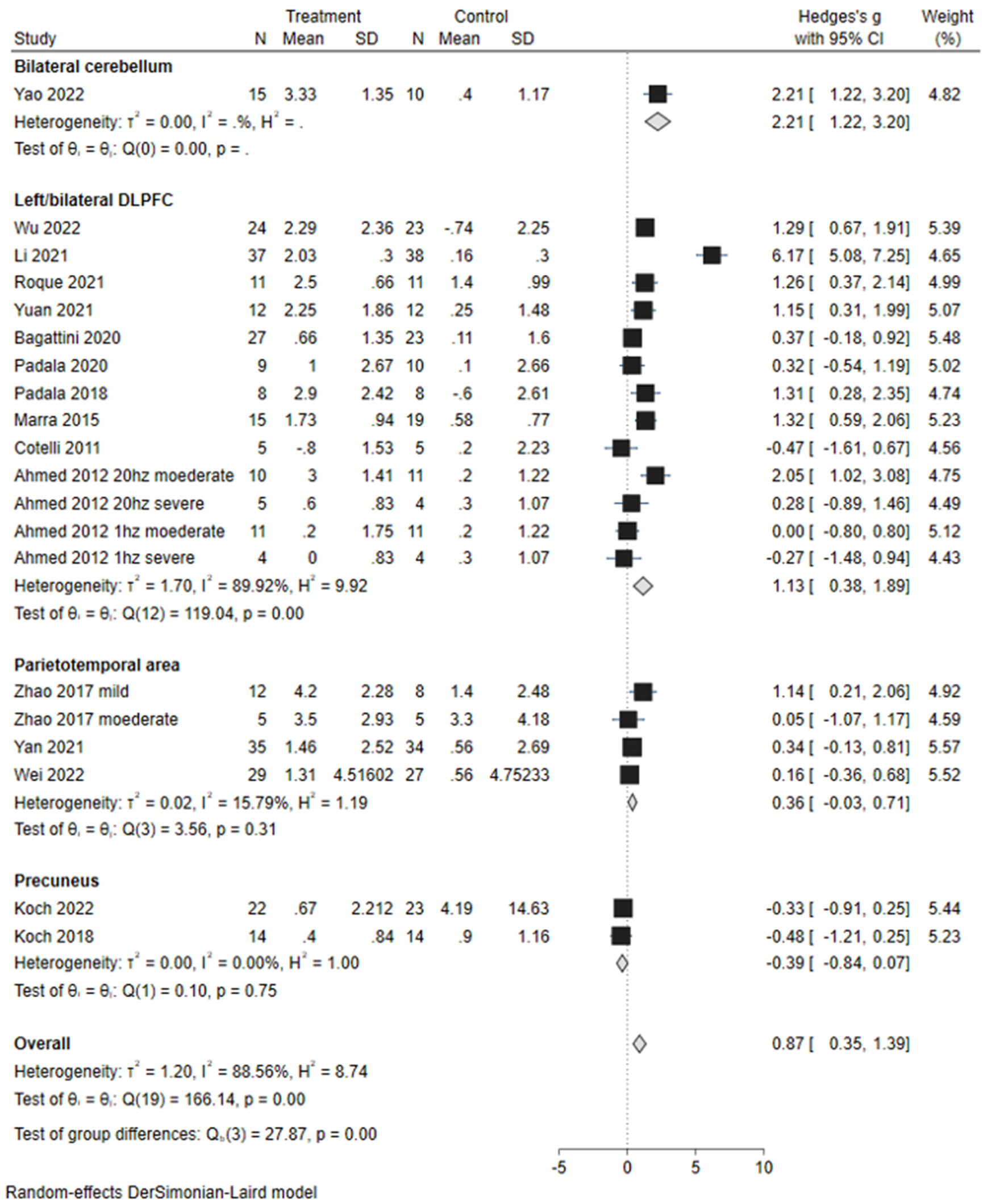

Supplement: Supplementary Figure 9 — Forest plot: the subgroup analysis of the stimulation sites in the short-term effects. [file Image_9.TIF]

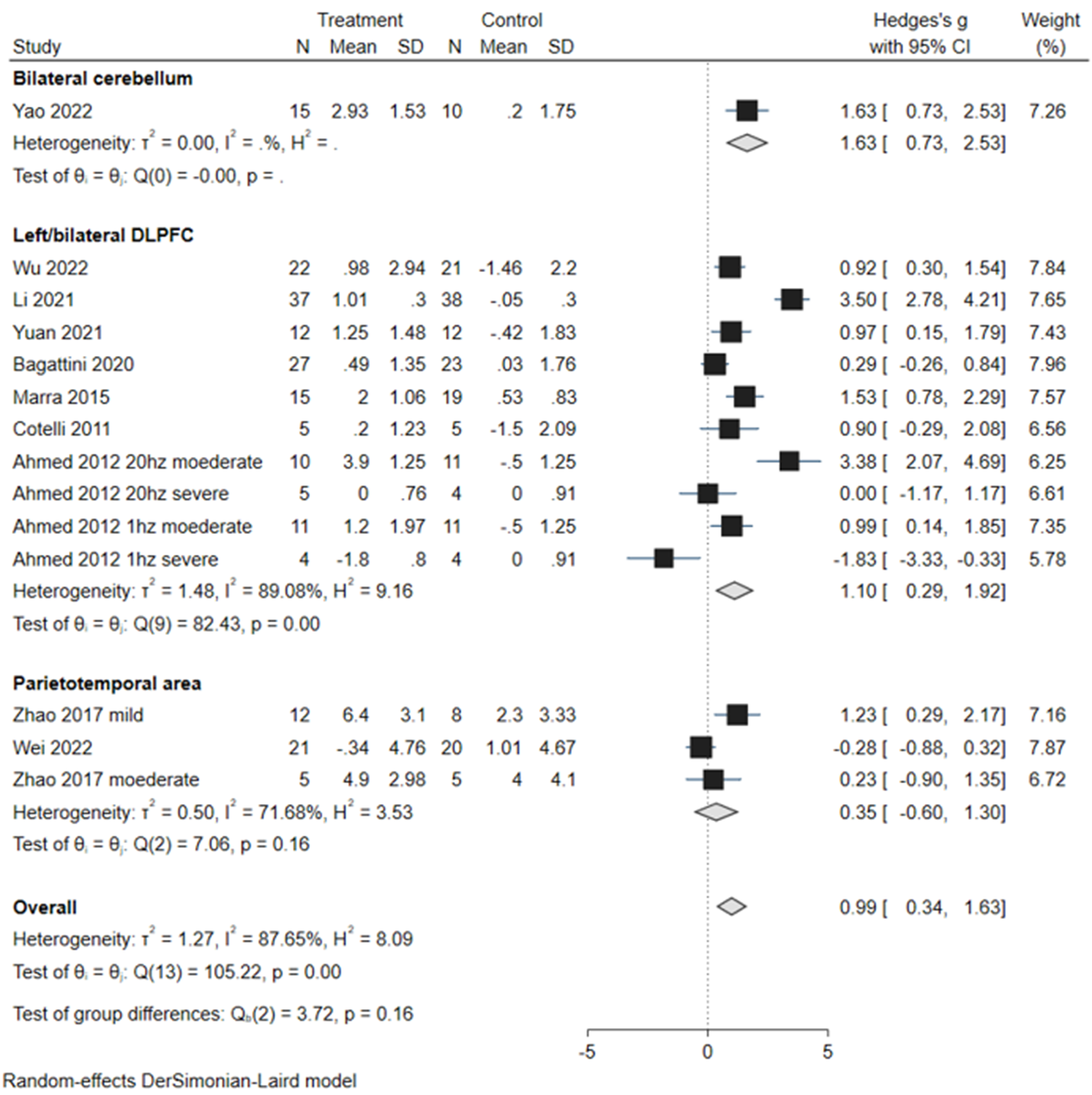

Supplement: Supplementary Figure 10 — Forest plot: the subgroup analysis of the stimulation sites in the long-lasting effects. [file Image_10.TIF]

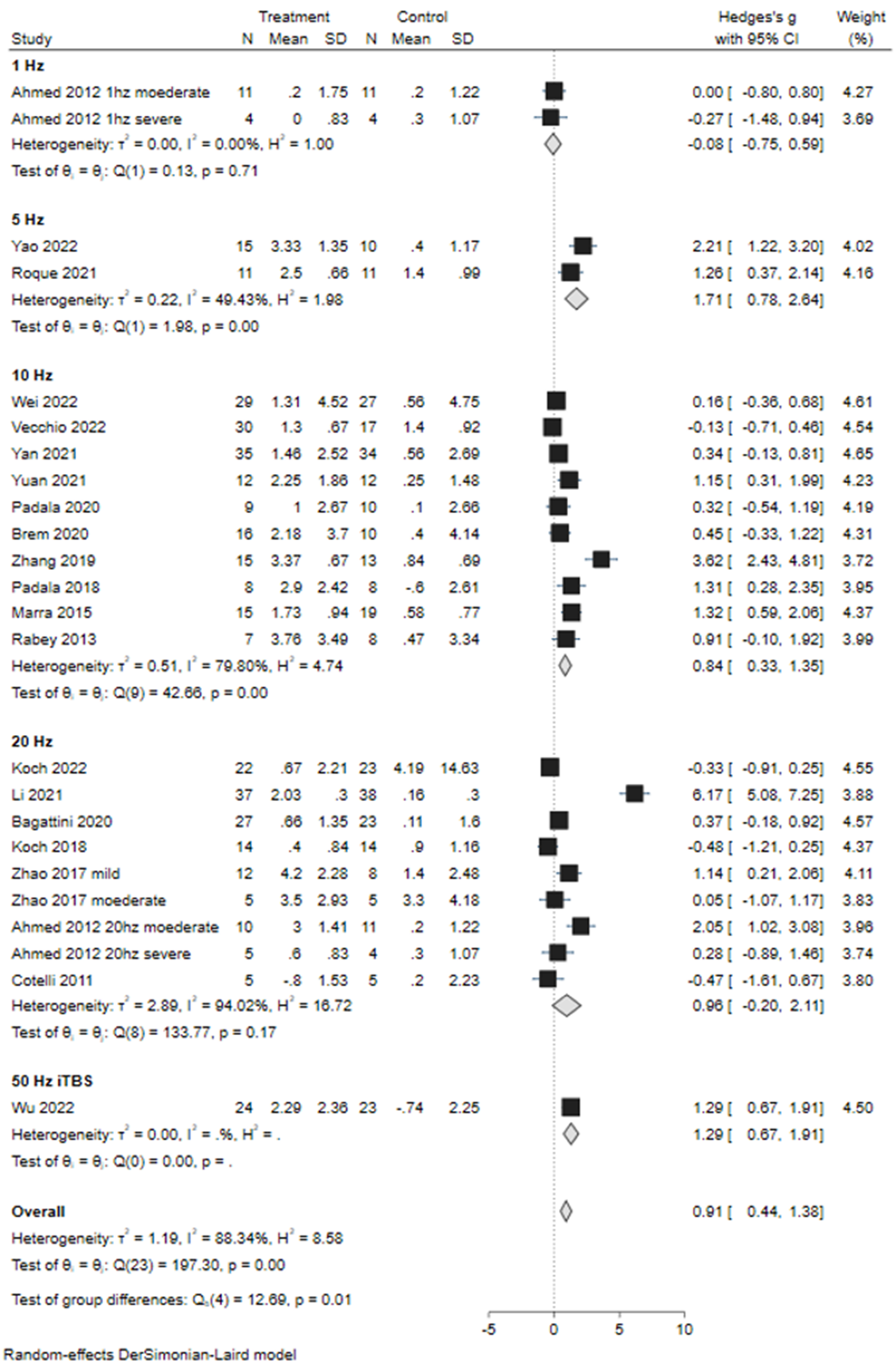

Supplement: Supplementary Figure 11 — Forest plot: the subgroup analysis of the TMS frequency in the short-term effects. [file Image_11.TIF]

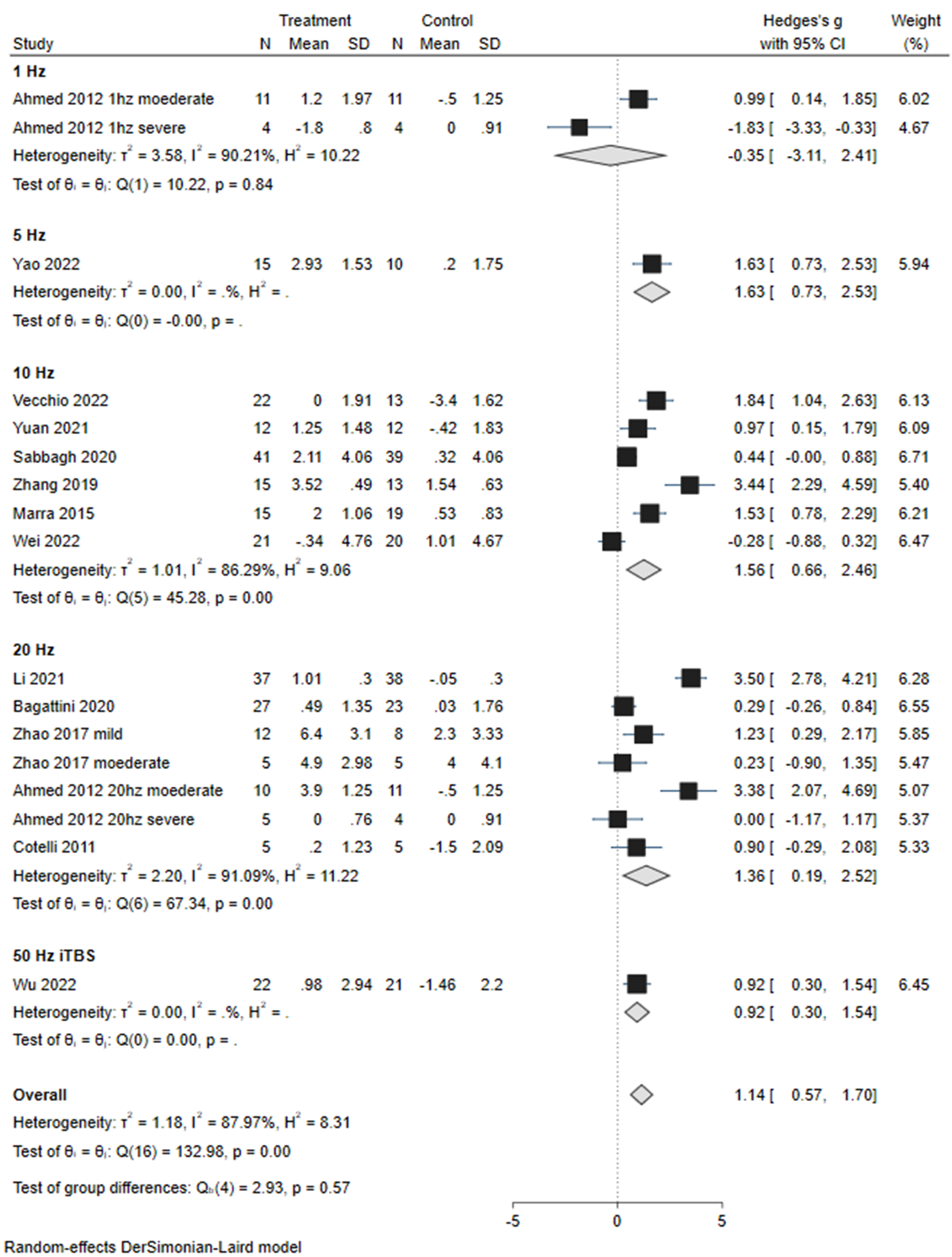

Supplement: Supplementary Figure 12 — Forest plot: the subgroup analysis of the TMS frequency in the long-lasting effects. [file Image_12.TIF]

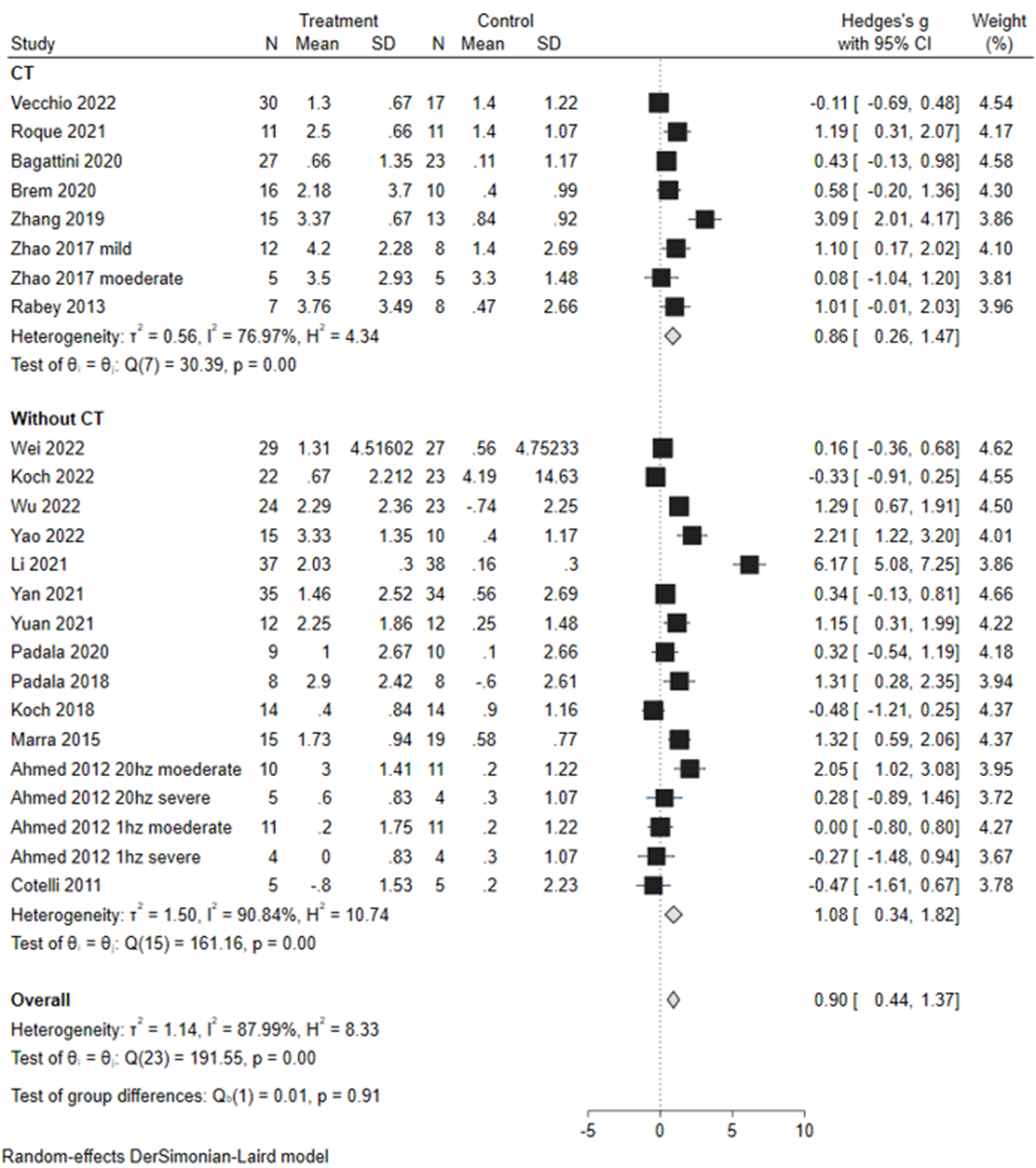

Supplement: Supplementary Figure 13 — Forest plot: the subgroup analysis of the with/without CT in the short-term effects. [file Image_13.TIF]

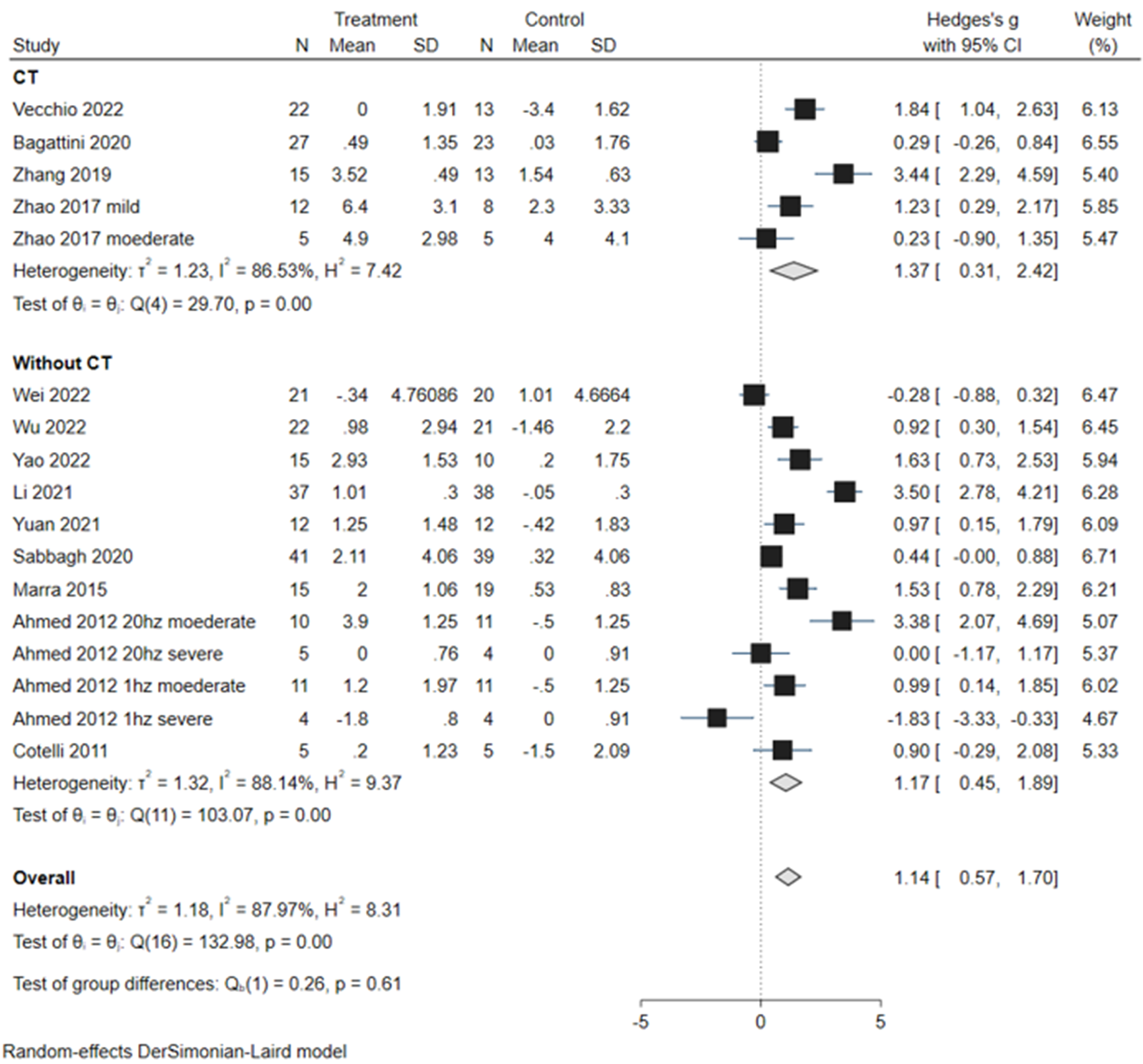

Supplement: Supplementary Figure 14 — Forest plot: the subgroup analysis of the with/without CT in the long-lasting effects. [file Image_14.TIF]

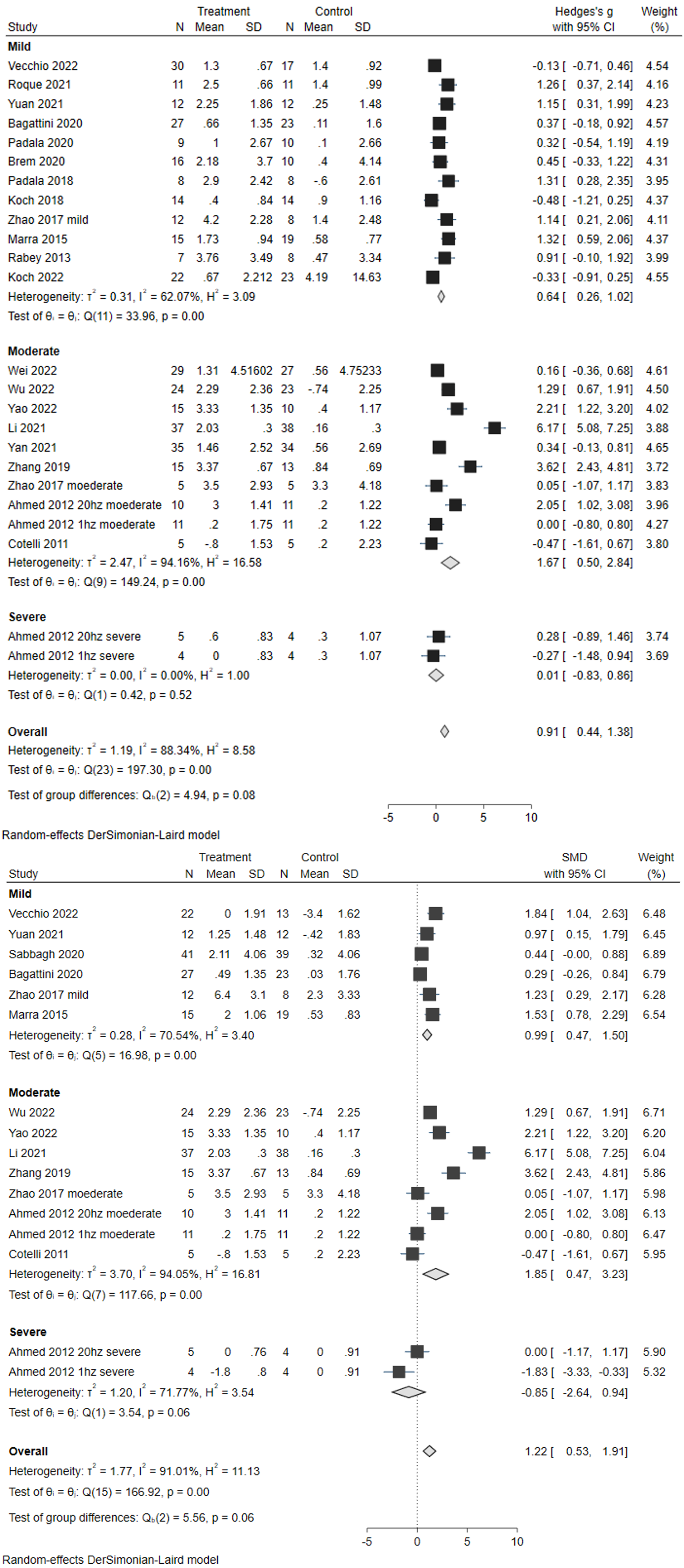

Supplement: Supplementary Figure 15 — Forest plot: the subgroup analysis of patient characteristics in the short-term effects (Above) and in the long-lasting effects (Below). [file Image_15.TIF]
